# Investigating Parkinson’s disease risk across farming activities using data mining and large-scale administrative health data

**DOI:** 10.1038/s41531-024-00864-2

**Published:** 2025-01-08

**Authors:** Pascal Petit, François Berger, Vincent Bonneterre, Nicolas Vuillerme

**Affiliations:** 1 Univ. Grenoble Alpes, AGEIS, 38000 Grenoble, France; 2 Univ. Grenoble Alpes, INSERM, Unit 1205, Braintech Lab, 38000 Grenoble, France; 3 Univ. Grenoble Alpes, CNRS, UMR 5525, VetAgro Sup, Grenoble INP, CHU Grenoble Alpes, TIMC, 38000 Grenoble, France; 4 CHU Grenoble Alpes, Centre Régional de Pathologies Professionnelles et Environnementales, 38000 Grenoble, France; 5 Institut Universitaire de France, 75000 Paris, France

**Keywords:** Epidemiology, Parkinson's disease, Risk factors

## Abstract

The risk of Parkinson’s disease (PD) associated with farming has received considerable attention, in particular for pesticide exposure. However, data on PD risk associated with specific farming activities is lacking. We aimed to explore whether specific farming activities exhibited a higher risk of PD than others among the entire French farm manager (FM) population. A secondary analysis of real-world administrative insurance claim data and electronic health/medical records (TRACTOR project) was conducted to estimate PD risk for 26 farming activities using data mining. PD cases were identified through chronic disease declarations and antiparkinsonian drug claims. There were 8845 PD cases among 1,088,561 FMs. The highest-risk group included FMs engaged in pig farming, cattle farming, truck farming, fruit arboriculture, and crop farming, with mean hazard ratios (HRs) ranging from 1.22 to 1.67. The lowest-risk group included all activities involving horses and small animals, as well as gardening, landscaping and reforestation companies (mean HRs: 0.48–0.81). Our findings represent a preliminary work that suggests the potential involvement of occupational risk factors related to farming in PD onset and development. Future research focusing on farmers engaged in high-risk farming activities will allow to uncover potential occupational factors by better characterizing the farming exposome, which could improve PD surveillance among farmers.

## Introduction

Parkinson’s disease (PD) is the most common muscular functioning disorder and the second most common neurodegenerative disease after Alzheimer’s disease (AD), which affects millions of adults worldwide^1–8^. PD has a long prodromal phase (up to several decades), during which many possible protective and risk factors can contribute to the onset, development, and/or progression of PD^4,9^. PD is a multifactorial disorder characterized by complex interactions between genetic, behavioral, and environmental factors^1,4,6,10,11^. Most individuals diagnosed with PD have no family history of the disease, since genetics only accounts for 5–10% of the PD case^1,3,4^. Some protective (e.g., cigarette smoking)^4,6,8,12,13^ and risk factors (e.g., alcohol consumption)^4^ are modifiable, while others are not (e.g., aging and being a male)^6,14^. Identifying and understanding these factors is crucial to developing effective prevention strategies and interventions^9,11^.

The PD risk associated with farming activities has received considerable attention, in particular for pesticide exposure^2,7,15^, which involves low-dose cocktail effects^16^. The exposure to pesticides and other stressors (agricultural exposome^17^) strongly depends on the type of crops and livestock. Because of the broad range of farming activities, it is essential to study specific farming types that could act as a proxy for distinct agricultural exposome, as highlighted by several recent works^15,17–19^. However, to our knowledge, studies examining the association between PD risk and specific farming activities are limited, with investigations typically covering only 4–18 different types of crops and animal farming^18–26^. Most of these studies are not population-based and rely on a case-control design, which is prone to recall bias. They also focus on limited geographical areas, with few sex-specific analyses^1,7,19^. Furthermore, the existing research has primarily been conducted in France (*n* = 5)^18–22^, the US (*n* = 2)^25,26^, and Canada (*n* = 2)^23,24^. French studies include two nationwide ecological studies^18,20^ as well as one case–control study^22^, one cross-sectional study^21^, and one prospective cohort^19^, which were limited geographically. To complement these studies, large-scale administrative health data can be used. Administrative health data does not require additional time or effort for data collection and is population-based, with large sample sizes spanning multiple years^27–29^.

This study aimed, for the first time, to explore, using data mining and nationwide administrative health data, whether and to what extent specific farming activities exhibited a higher risk of PD than others, both overall and by sex category, among the entire French farm manager (FM) population. A FM refers to an individual who owns and/or oversees a farm (farm/company managers, owners, and self-employed persons) while performing a broad range of activities directly in the field (e.g., pesticide application, tractor driving, harvesting, or milking)^30^.

## Results

### Population characteristics

There were 1,088,561 FMs, among which 8845 FMs were identified as PD cases in the main analysis (Supplementary Table 1), resulting in an incidence rate of 0.28 [95% CI: 0.27–0.29] PD cases per 1000 persons-years, with 0.40 [0.38–0.41] cases per 1000 persons-years for males and 0.24 [0.23–0.25] cases per 1000 persons-years for females (Supplementary Table 2). All PD cases had a drug reimbursement, while only 3034 (34%) had a LTI (long-term illness) declaration and 317 (4%) had an ODC declaration (occupational disease covered under workers’ compensation statutes) for PD induced by pesticide exposure (Supplementary Table 3). Overall, FMs with PD were older than FMs without PD (mean age of 55 years old vs. 47 years old) (Table 1).

**Table 1:** Baseline characteristics of the study population, TRACTOR project, France, 2012–2016

	FM without PD (*n* = 1,079,716)	FM with PD (*n* = 8845)
*Main characteristics*	*n* (%)	*n* (%^a^) [overall %^b^]
Sex		
Male	745,798 (69.1)	5500 (61.2) [0.73]
Female	333,918 (30.9)	3345 (38.8) [0.99]
Age at baseline: 2012/01/01 (years)		
Mean (SD) [min-max]	46.5 (14.1) [18–96]	55.8 (12.6) [34–93]
Family status		
Single	465,923 (43.2)	3087 (34.9) [0.66]
As a couple	613,793 (56.8)	5758 (65.1) [0.93]
First year of the farm’s establishment		
Median (IQR)	1994 (39)	1990 (39.9)
Farm surface (expressed in hectares)		
Median (IQR)	1.64 (14.0)	1.93 (14.1)
Farm location (region)		
Auvergne-Rhône-Alpes	116,588 (10.8)	840 (9.5) [0.72]
Bourgogne-Franche-Comté	65,323 (6.05)	541 (6.12) [0.82]
Bretagne	80,791 (7.48)	511 (5.78) [0.63]
Centre - Val de Loire	49,424 (4.58)	410 (4.64) [0.82]
Corse	5296 (0.49)	30 (0.34) [0.56]
Grand Est	81,022 (7.5)	732 (8.28) [0.90]
Hauts-de-France	47,908 (4.44)	490 (5.54) [1.01]
Île-de-France	13,978 (1.29)	106 (1.2) [0.75]
Normandie	79,917 (7.4)	721 (8.15) [0.89]
Nouvelle-Aquitaine	178,771 (16.6)	1612 (18.2) [0.89]
Occitanie	165,387 (15.3)	1438 (16.3) [0.86]
Provence-Alpes-Côte d’Azur	113,201 (10.5)	852 (9.63) [0.75]
Pays de la Loire	82,110 (7.6)	562 (6.35) [0.68]
Number of farms		
1 farm	1,053,455 (97.6)	8655 (97.9) [0.81]
>1 farm	26,261 (2.43)	190 (2.15) [0.72]
Farm type (farm clustering)		
Individual farm	750,001 (69.5)	6587 (74.5) [0.87]
Farm with work partners	329,715 (30.5)	2258 (25.5) [0.68]
Partner work status		
Perform task to help farm manager	126,923 (11.8)	1328 (15) [1.04]
Do not perform task to help farm manager	952,793 (88.2)	7517 (85) [0.78]
Number of associates		
0	829,228 (76.8)	7004 (79.2) [0.84]
≥ 1	250,488 (23.2)	1841 (20.8) [0.73]
Secondary farming activity^†^		
No secondary farming activity performed in in parallel to a main activity	678,780 (62.9)	7810 (88.3) [1.14]
At least one farming activity performed in in parallel to a main activity	400,936 (37.1)	1035 (11.7) [0.26]
Number of different yearly activities performed		
Only one activity during the observation period	971,334 (90.0)	8116 (91.8) [0.83]
Two activities during the observation period	101,735 (9.42)	704 (7.96) [0.69]
At least three activities during the observation period	6647 (0.62)	25 (0.28) [0.37]
Lack of job security		
Has never been unemployed during the observation period	1,077,605 (99.8)	8838 (99.9) [0.81]
Has been unemployed during the observation period	2111 (0.2)	7 (0.08) [0.33]
Median yearly insurance premium (euros)		
Median (IQR)	5166 (40818)	4592 (40877)
Employees		
No employee	783,007 (72.5)	7137 (80.7) [0.90]
At least one employee	296,709 (27.5)	1708 (19.3) [0.57]
Work status		
Working as a farm manager	814,203 (75.4)	6544 (74) [0.80]
Working as a solidarity contributor*	265,513 (24.6)	2301 (26) [0.86]
Retirement status		
Did not retired before the disease diagnosis or end of follow-up	761,653 (70.5)	2808 (31.7) [0.37]
Retired before the disease diagnosis or end of follow-up	318,063 (29.5)	6037 (68.3) [1.86]
Pre-existing disability		
Did not become disabled before the disease diagnosis or end of follow-up	1,075,485 (99.6)	8657 (97.9) [0.80]
Became disabled before the disease diagnosis or end of follow-up	4231 (0.39)	188 (2.13) [4.25]
Number of pre-existing comorbidities (long term illness)		
0 comorbidity before the disease diagnosis or end of follow-up	704,914 (65.3)	4602 (52) [0.65]
1 comorbidity before the disease diagnosis or end of follow-up	214,432 (19.9)	2464 (27.9) [1.14]
>1 comorbidity before the disease diagnosis or end of follow-up	160,370 (14.9)	1779 (20.1) [1.10]

Note: *FM* farm manager, *IQR* interquartile range, *PD* Parkinson’s disease, *SD* arithmetic standard deviation.

^a^Percentage of PD cases among all FMs with PD.

^b^Percentage of PD cases among all FMs with the given characteristics.

^†^A secondary farming activity is defined as a farming activity (e.g., grassland farming) that a FM can perform in addition to its main activity (e.g., ovine farming). The nature of the secondary activity is however unknown.

*FMs who farm on a small surface (<12.5 ha) or who works less than 1200 h/year.

### PD risk associated with farming activities

Associations varied by types of crops and animal farming (Table 2, and Supplementary Table 4). Elevated HRs were observed for fruit arboriculture (HR = 1.35 [1.08–1.68]), pig farming (HR = 1.39 [1.16–1.68]), dairy farming (HR = 1.48 [1.40–1.57]), mixed cattle farming (HR = 1.58 [1.41–1.46]), and crop farming (HR = 1.67 [1.53–1.82]) (Table 2). Modestly elevated HR were found for cow farming (HR = 1.22 [1.14–1.30]), truck farming (HR = 1.22 [1.09–1.36]), and unspecified and mixed farming (HR = 1.09 [1.02–1.16]). A positive trend was observed in viticulture (HR = 1.07 [0.94–1.21]) and ovine and caprine farming (HR = 1.07 [0.96–1.20]) (Table 2).

**Table 2:** Risks of Parkinson’s disease by agricultural activity, TRACTOR project, France, 2012–2016

Farming activity	Sex	Study population no. (%)	PD^*^ no. (%)	Unexposed PD no.	HR [95% CI]^†^	*p*	adj. *p*
Crop farming (e.g., wheat, corn, and industrial grower)	Both sexes	317,458 (29.2)	2998 (33.9)	5847	**1.67 [1.53–1.82]**	**5.0e−30**	**2.7e−29**
	Female	106,016 (31.4)	1238 (37.0)	2107	**1.74 [1.50–2.02]**	**3.4e−13**	**4.3e−13**
	Male	211,442 (28.1)	1760 (32.0)	3740	**1.67 [1.50–1.87]**	**3.5e−20**	**9.6e−20**
Mixed cattle farming	Both sexes	31,309 (2.88)	324 (3.66)	8521	**1.58 [1.41–1.76]**	**1.3e−15**	**2.1e−14**
	Female	8206 (2.43)	116 (3.47)	3229	**1.72 [1.43–2.07]**	**1.1e−08**	**3.0e−8**
	Male	23,103 (3.08)	208 (3.78)	5292	**1.47 [1.28–1.69]**	**7.0e−8**	**1.5e−07**
Dairy farming	Both sexes	162,906 (15.0)	1412 (16.0)	7433	**1.48 [1.40-1.57]**	**5.8e−40**	**3.2e−39**
	Female	50,385 (14.9)	532 (15.9)	2813	**1.45 [1.32–1.59]**	**2.4e−14**	**3.6e−14**
	Male	112,521 (15.0)	880 (16.0)	4620	**1.45 [1.35–1.56]**	**6.5e−23**	**2.4e−22**
Pig farming	Both sexes	13,760 (1.26)	111 (1.25)	8734	**1.39 [1.16–1.68]**	**5.2e−04**	**1.5e−03**
	Female	3948 (1.17)	42 (1.26)	3303	**1.62 [1.19–2.20]**	**1.9e−03**	**4.4e−03**
	Male	9812 (1.31)	69 (1.25)	5431	1.25 [0.99–1.59]	0.06	0.07
Cow farming	Both sexes	113,244 (10.4)	1015 (11.5)	7830	**1.22 [1.14–1.30]**	**6.0e−9**	**2.0e−7**
	Female	33,603 (9.96)	371 (11.1)	2974	**1.23 [1.11–1.37]**	**1.6e−04**	**4.8e−04**
	Male	79,641 (10.6)	644 (11.7)	4856	**1.20 [1.10–1.30]**	**2.0e−5**	**1.1e−04**
Truck farming, floriculture/flower-growing	Both sexes	43,684 (4.01)	312 (3.53)	8533	**1.22 [1.09–1.36]**	**6.5e−04**	**4.2e−03**
	Female	13,350 (3.96)	122 (3.65)	3223	**1.32 [1.10–1.58]**	**3.0e−3**	**9.6e−03**
	Male	30,334 (4.04)	190 (3.45)	5310	1.16 [1.00–1.34]	**0.05**	0.06
Fruit arboriculture	Both sexes	25,090 (2.30)	201 (2.27)	8644	**1.35 [1.08–1.68]**	**8.9e−03**	**0.03**
	Female	7981 (2.37)	67 (2.00)	3278	1.34 [0.91–2.02]	0.12	0.15
	Male	17,109 (2.28)	134 (2.44)	5366	**1.36 [1.04–1.79]**	**0.02**	**0.05**
Unspecified and mixed farming (e.g., polyculture, mixed farming, diversified farming)	Both sexes	129,013 (11.9)	1013 (11.5)	7832	**1.09 [1.02–1.17]**	**9.2e−03**	**0.04**
	Female	39,914 (11.8)	396 (11.8)	2949	1.11 [1.00–1.23]	0.06	0.09
	Male	89,099 (11.9)	617 (11.2)	4883	1.07 [0.98–1.16]	0.13	0.17
Viticulture	Both sexes	122,713 (11.3)	1169 (13.2)	7676	1.07 [0.94–1.21]	0.3	0.31
	Female	43,370 (12.9)	492 (14.7)	2853	0.89 [0.72–1.11]	0.24	0.30
	Male	79,343 (10.6)	677 (12.3)	4823	**1.23 [1.05–1.43]**	**9.2e−03**	**0.03**
Ovine and caprine farming	Both sexes	49,061 (4.51)	330 (3.73)	8515	1.07 [0.96–1.20]	0.2	0.65
	Female	17,529 (5.20)	118 (3.53)	3227	0.96 [0.80–1.15]	0.64	0.82
	Male	31,532 (4.20)	212 (3.85)	5288	1.15 [1.00–1.32]	**0.05**	0.65
Wood production (e.g., lopping)	Both sexes	11,431 (1.05)	55 (0.62)	8790	1.11 [0.85–1.44]	0.46	0.79
	Female	303 (0.09)	2 (0.06)	3343	Not calculated		
	Male	11,128 (1.48)	53 (0.96)	5447	1.18 [0.90–1.55]	0.23	0.52
Garden center/tree nursery	Both sexes	5418 (0.50)	33 (0.37)	8812	1.09 [0.78–1.54]	0.61	0.79
	Female	1438 (0.43)	6 (0.18)	3339	0.66 [0.30–1.48]	0.31	0.79
	Male	3980 (0.53)	27 (0.49)	5473	1.28 [0.88–1.87]	0.2	0.79
Poultry and rabbit farming	Both sexes	25,863 (2.38)	124 (1.40)	8721	0.90 [0.75–1.07]	0.24	0.92
	Female	10,196 (3.02)	53 (1.58)	3292	0.87 [0.66–1.14]	0.3	0.92
	Male	15,667 (2.09)	71 (1.29)	5429	0.91 [0.72–1.15]	0.43	0.92
Unspecified large animal farming (e.g., ostrich, llama)	Both sexes	3020 (0.28)	16 (0.18)	8829	1.15 [0.70–1.87]	0.58	0.75
	Female	1458 (0.43)	6 (0.18)	3339	0.82 [0.37–1.83]	0.63	0.75
	Male	1562 (0.21)	10 (0.18)	5490	1.45 [0.78–2.70]	0.24	0.75
Agricultural work companies (e.g., pesticide applications, harvest reaping)	Both sexes	15,635 (1.44)	60 (0.68)	8785	0.90 [0.70–1.16]	0.4	1.0
	Female	1887 (0.56)	7 (0.21)	3338	0.77 [0.37–1.61]	0.49	1.0
	Male	13,748 (1.83)	53 (0.96)	5447	0.94 [0.72–1.23]	0.65	1.0
Sylviculture/forestry (e.g., thinning, pruning)	Both sexes	2166 (0.20)	16 (0.18)	8829	1.10 [0.68–1.80]	0.7	0.96
	Female	361 (0.11)	6 (0.18)	3339	1.57 [0.71–3.50]	0.27	0.68
	Male	1805 (0.24)	10 (0.18)	5490	1.00 [0.54–1.85]	0.99	1.0
Company representative/authorized representative	Both sexes	1935 (0.18)	9 (0.10)	8836	1.22 [0.64–2.35]	0.55	0.64
	Female	1500 (0.45)	7 (0.21)	3338	1.20 [0.57–2.52]	0.63	0.69
	Male	435 (0.06)	2 (0.04)	5498	Not calculated		
Unspecified specialized farming (e.g., herbs, mushrooms)	Both sexes	6671 (0.61)	29 (0.33)	8816	0.82 [0.57–1.18]	0.28	0.6
	Female	2438 (0.72)	9 (0.27)	3336	0.63 [0.33–1.22]	0.17	0.5
	Male	4233 (0.56)	20 (0.36)	5480	0.93 [0.60–1.45]	0.75	0.89
Stationary sawmill (e.g., edging, trimming, decking, debarking)	Both sexes	791 (0.07)	6 (0.07)	8839	1.24 [0.56–2.76]	0.6	0.72
	Female	55 (0.02)	0 (0)	3345	Not calculated		
	Male	736 (0.10)	6 (0.11)	5494	1.41 [0.63-3.14]	0.4	0.6
Shellfish farming (e.g., oyster farming, scallop aquaculture)	Both sexes	3825 (0.35)	12 (0.14)	8833	0.65 [0.37–1.15]	0.14	0.59
	Female	736 (0.22)	3 (0.09)	3342	0.71 [0.23–2.21]	0.56	0.65
	Male	3089 (0.41)	9 (0.16)	5491	0.67 [0.35–1.29]	0.23	0.59
Salt works/salt evaporation pond	Both sexes	980 (0.09)	3 (0.03)	8842	0.59 [0.19–1.83]	0.36	0.48
	Female	220 (0.07)	1 (0.03)	3344	Not calculated		
	Male	760 (0.10)	2 (0.04)	5498	Not calculated		
Gardening, landscaping and reforestation companies	Both sexes	49,177 (4.52)	150 (1.70)	8695	**0.81 [0.69–0.95]**	**0.01**	**0.05**
	Female	2562 (0.76)	10 (0.30)	3335	0.91 [0.49–1.70]	0.77	0.88
	Male	46,615 (6.20)	140 (2.55)	5360	0.88 [0.74–1.04]	0.13	0.27
Unspecified small animal farming (e.g., frogs, snails, bees)	Both sexes	19,984 (1.84)	54 (0.61)	8791	**0.51 [0.39–0.67]**	**7.7e−07**	**1.9e−05**
	Female	8632 (2.56)	18 (0.54)	3327	**0.39 [0.25–0.62]**	**7.3e−05**	**3.3e−04**
	Male	11,352 (1.51)	36 (0.65)	5464	**0.59 [0.43–0.82]**	**1.7e−03**	**2.7e−03**
Stud farming	Both sexes	17,269 (1.59)	53 (0.60)	8792	**0.48 [0.37–0.63]**	**1.3e−07**	**7.6e−07**
	Female	7486 (2.22)	13 (0.39)	3332	**0.28 [0.16–0.48]**	**4.8e−06**	**1.4e−05**
	Male	9783 (1.30)	40 (0.73)	5460	**0.64 [0.47–0.87]**	**4.5e−03**	**7.9e−03**
Training, dressage, riding clubs	Both sexes	14,979 (1.38)	30 (0.34)	8815	**0.49 [0.35–0.71]**	**1.2e−04**	**2.5e−03**
	Female	6687 (1.98)	7 (0.21)	3338	**0.29 [0.14–0.60]**	**9.6e−04**	**4.8e−03**
	Male	8292 (1.10)	23 (0.42)	5477	**0.63 [0.42-0.95]**	**0.03**	**0.05**
Rural craftsperson (e.g., mason, mechanics)	Both sexes	7696 (0.71)	5 (0.06)	8840	**0.14 [0.06–0.34]**	**1.3e−05**	**2.0e−5**
	Female	283 (0.08)	0 (0)	3345	Not calculated		
	Male	7413 (0.99)	5 (0.09)	5495	**0.15 [0.06–0.36]**	**2.1e−05**	**3.0e−5**

Abbreviations: *PD* Parkinson’s disease, *HR* hazard ratio, *m* number of exposed PD cases, *p* p-value, *adj. p* *p*-value adjusted using the Benjamini-Hochberg approach.

^*^The percentages in brackets refer to the ratio of exposed PD cases in the study population to the total number of PD cases in the overall population.

^†^Main analysis: adjusted for sex (for “both sexes” only), age, the first year of the farm’s establishment, farm surface, number of associates, unemployment status, total number of farms, family status, partner work status, farm location, number of comorbidities, and having a secondary farming activity.

By contrast, the lowest-risk group included FMs engaged in gardening, landscaping and reforestation companies (HR = 0.81 [0.69–0.95]), small animal farming (HR = 0.51 [0.39–0.67]), training, dressage and riding clubs (HR = 0.49 [0.35–0.71]), stud farming (HR = 0.48 [0.37–0.63]), and rural craftsperson (HR = 0.14 [0.06–0.34]) (Table 2). Most activities did not exhibit any sex difference, with the exception of viticulture and stud farming (Supplementary Table 5). In viticulture, male FMs (HR = 1.23 [1.05–1.43]) had a more elevated HR than females (HR = 0.89 [0.72–1.11]) (Supplementary Table 5).

**Fig. 1:**
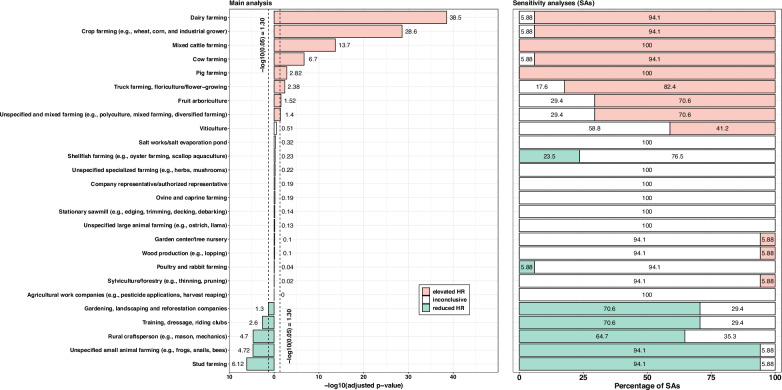
Farming activities and risks of PD, TRACTOR project, France, 2012–2016. Comparison of primary and sensitivity analyses.

All sensitivity analyses yielded similar results to the main analysis (Fig. 1, Supplementary Table 4). There were a few exceptions. When excluding PD cases diagnosed in 2012 (SA1), the positive association with fruit arboriculture disappeared (Supplementary Table 4). Regarding the sensitivity analysis for which PD cases were solely identified using ODC declaration for PD induced by pesticide exposure (SA2), positive associations were found only for pig farming (HR = 2.47 [1.31–4.64]), mixed cattle farming (HR = 2.14 [1.37–3.33]), viticulture (HR = 1.65 [1.15–2.38]), unspecified and mixed farming (HR = 1.42 [1.05–1.93]), and dairy farming (HR = 1.32 [0.99–1.77]) (Supplementary Table 4).

The bar plot consists of two panels. The left panel shows the main analysis, with each bar representing the negative log-transformed adjusted *p*-value (*x*-axis) for each farming activity (*y*-axis). Red bars indicate a higher PD risk (hazard ratio > 1), green bars a lower PD risk (hazard ratio < 1), and white bars denote activities where the hazard ratio 95% confidence interval includes 1. Vertical dashed lines mark the −log10 (0.05) threshold. The right panel presents a stacked bar plot showing the percentage of sensitivity analyses out of 17 (*x*-axis) in which each farming activity (*y*-axis) was associated with higher (red), lower (green), or unchanged PD risk (white). Please refer to the Supplementary materials for more details about sensitivity analyses.

All analyses adjusted for sex (for “both sexes” only), age, farm establishment year, median farm surface, number of associates, unemployment status, farm count, family status, partner employment status, farm location, number of comorbidities, and performing a secondary activity.

## Discussion

For the first time, the association between PD risk and multiple farming activities was examined, overall and by sex category, in the entire French FM population using data mining and large-scale real-world administrative health data. As suspected, the association between the risk of PD and farming highly varied depending on the activity considered, with a 3.4-fold range observed between the lowest and highest HRs. The highest-risk group included FMs engaged in pig farming, cattle farming, fruit arboriculture, viticulture, truck farming, and crop farming, while the lowest-risk group included all FMs engaged in activities involving horses and unspecified small animal farming, but also gardening, landscaping and reforestation companies. This PD risk heterogeneity suggests that part of the risk may possibly be attributable to specific farming practices, with a few sex-specific PD risks that could denote differences in occupational exposures and tasks between males and females, as previously suggested^15^. Potential hormonal disparities may also have played a role because several studies suggest a possible protective effect of female sex hormones (especially estrogen)^31–34^. However, this study is only a preliminary work, which highlights the need for further research.

FMs with PD were older than FMs without PD, which is consistent with the literature as PD affects people late in life, usually starting after 50 years old^2,5,7,35^. In line with previous works, the PD incidence rate increased with age, was higher for males than females^4,5,7,19,21^, and was consistent with those of the entire French population^36^ and other studies^4,6,7,23^.

During farming activities, FMs can be exposed to numerous environmental co-occurring contaminants (agricultural exposomes)^29^, some of which could be implicated in PD. Of particular interest are pesticides, which are used to protect crops and livestock from pest infestations and diseases^16,37^. Numerous studies have reported an association between pesticide exposure and PD, but results are often inconsistent and limited, in particular for specific pesticide compounds, probably because of the large number of different pesticides each FM used throughout his/her career^1,2,8,12,15,18,35^. The paraquat herbicide^19,38–46^, 2,4-D herbicide^7,44,47^, triazine (atrazine) herbicides^2,44,48,49^, rotenone insecticide^19,40,41,45,50^, fipronil insecticide^2,44,51^, organophosphates (OPs)^50,52–55^, organochlorines^44,53,54,56,57^, pyrethroids^40,44,49^, as well as the fungicides maneb^19,39,46^, mancozeb^12,19^, and ziram^7,12,19,46^ are among the most frequently incriminated pesticides.

While the pesticides used may sometimes be common between farming types, their amount, frequency, intensity, and mode of application also strongly vary and change over time^18,19,21^. For instance, there is a higher use of pesticides in truck farming, crop farming, fruit arboriculture, and viticulture than in other farming activities^18^. Farms specialized in orchards rank first in terms of insecticide use, whereas viticulture uses mostly fungicides, and cereal crop farming employs insecticides, herbicides, and fungicides, but with lower frequency and intensity^18,22^. Regarding the treatment of ectoparasites in animal husbandry, OP insecticides have preferentially been used on animals since the 1970s^19^, replacing the organochlorine insecticide lindane, before being substituted by pyrethroids and sometimes oral medications only (ivermectine). Hence, the farming type is a proxy of agricultural exposure that is easier to assess than specific pesticides^21^ and may better reflect the agricultural exposome^29^, for which pesticide association plays a pivotal role, with a low-dose cocktail effect^16^.

Several French studies reported an association between PD and pesticide use and pesticide expenditures in vineyards^18,22^ or in regions with a greater presence of vineyards, cereal farming, fruit crops, fresh vegetable farming, and pig farming^20,21^. Another French study (prospective cohort AGRICAN), including both FMs and farm workers from 13 French departments using self-administered questionnaires, reported that lifelong pesticide use was associated with an increased risk of PD in all types of activities (e.g., cattle, crop farming)^19^. This study, which included 1732 PD cases, also found positive associations between PD and farmers exposed to insecticides on animals, which were particularly strong for pig farming^19^. A Canadian case-control study with 403 PD cases reported a higher PD risk for ever-occupational contact with cattle and found a positive trend for swine^23^.

Regarding animal farming, FMs can also be exposed to pesticides from animal manure and feeds, in particular for cattle and pigs^14,23^, such as diethyltoluamide (35%), a repellent that has been implicated with PD^14^.

Interestingly, our findings for PD are different than those from a previous study conducted in the same population examining AD risk, in which only three farming activities were found to have a higher risk of AD (crop farming, fruit arboriculture, and viticulture), while all animal farming activities exhibited a lower risk of AD^30^. This could suggest that different exposome and potentially pesticides with different mechanisms could be involved. One possibility could be that the site of initial misfolding events differs between both neurodegenerative diseases. The Braak and dual-hit hypotheses posit that PD may first initiate in the olfactory structures (olfactory bulb) and the gut enteric nerves (gut-to-brain pathway)^9,58,59^. Hence, pesticides could gain access to the brain via the olfactory pathway or the vagus nerve, which could eventually trigger the spread of PD by a templating mechanism in a prion-like manner^9,59^. In contrast to PD, AD does not seem to be affected by the olfactory pathways^59,60^.

The cocktail effect, involving a low-dose mixture of pesticides, is now considered a pivotal physiopathological issue^16^. It makes classical epidemiological study very challenging. Recently, large-scale screening on dopaminergic cells demonstrated the involvement of 53 pesticides and the impact of specific mixtures on PD^16^. These in vitro studies are not fully relevant for the in vivo occupational situation, as it is not known if these compounds can reach the dopaminergic cells in the brain and also what the relevant dose is associated with neurodegeneration^61^. Our finding of specific farming activities associated with higher PD risk probably reflects specific mixtures of pesticides and environmental factors (agricultural exposomes). It suggests that “real-life screening” should be synergistically associated with this new field of diseases^61,62^. The chemical exposome^62,63^ needs to be better objectified in the future, using biological exposure monitoring (e.g., urine, blood, hair, or fat tissues) to help in the identification of specific mixtures of pesticides or environmental cocktails involved in PD neurodegeneration^61,62^.

The microbiota is today hypothesized to play a key role in PD^64^. Changes in gut microbiota can generate pro-inflammatory mediators and alpha-synuclein aggregation, which can be transported from the enteric nervous system to the brain via the vagus nerve (“Gut-Brain axis”). Some pesticides can disrupt the nasal and gut microbiome, cause mitochondrial dysfunction, affect lysomal function, or exert neurotoxic effects, in particular pesticides composed of heavy metals such as manganese, zinc, aluminum, copper, and mercury^5,14,18,49^. For instance, several fungicides contain metals, such as maneb (containing manganese), ziram (containing zinc), mancozeb (containing both zinc and manganese), or the Bordeaux mixture (containing copper), that were/are used in viticulture, crop, and fruit farming^18,65^. Exposure to some pathogenic bacteria can cause epigenetic changes in several PD risk factor genes^64^. The microbiome of farmers, and in particular livestock breeders, is completely altered by their specific occupational exposure, as shown by numerous studies conducted mainly in pig breeders^66–70^. Contact with animals during breeding activities is associated with changes in the dermal, nasal, and gut microbiota, as the microbiome of the upper respiratory track is then swallowed. In particular, it has been shown that the microbiome and resistome of farmers are very similar to those of their animals^66–70^.

Contact with animals can also expose FMs to zoonotic agents and endotoxins^23,71,72^, in particular Mycobacterium avium ss. paratuberculosis (MAP), which could start as an enteric infection via the vagus nerve, as hypothesized by one study^73^. Cattle farmers were also found to have a higher risk of both hypothyroidism and hyperthyroidism^17^ but also inflammatory bowel disease^74^, which have been associated with PD risk^64,75^.

Previous studies found evidence that alpha-synuclein’s activities can be affected by polystyrene and other particles found, for instance, in microplastics (MPs) and nanoplastics (NPs)^76,77^. MPs/NPs represent emerging environmental pollutants, with up to 430,000 tons of MPs potentially entering agricultural fields annually in Europe^78,79^. Pesticides and other toxic agents (e.g., infectious agents) can bind NPs that can act as carriers to facilitate blood-brain barrier crossing based on the “Trojan Horse” effect^80,81^. There are many possible sources of MPs/NPs contamination in agriculture. Direct contamination sources include plastic mulching, the cover of plastic greenhouses, or polymer-based fertilizers^79^. Indirect sources of contamination include the application of biosolids, the application of compost, or irrigation with waste-treated water^78,79^.

Our study is the first to focus on the entire FM population. Strengths of our study include the largest sample of FMs ever studied, its population-based and nationwide design, the large number of exposed PD cases, sex-specific analyses, adjustments to several potential confounders (sex, age, geographical area, farm surface), as well as the wide range of agricultural exposures with detailed information on 26 farming activities. In addition, contrary to case control studies or studies using declarative data, the administrative nature of our data (farming type as a means of assessing exposure) was not prone to recall bias, which could have led to exposure misclassification^1,18^. We chose to use a time-on-study approach instead of using age as timescale because several studies suggest that time-on-study models may be preferable, as they perform at least as well as left-truncated age scale models^82–84^. Additionally, they tend to be more robust against misspecification of the underlying time scale and generally offer better predictive ability^82–84^.

Our findings need to be considered in light of some limitations. First, the administrative nature of available data, which is characterized by a lack of accurate information on confounders and precise dates of disease onset^4,29^. Indeed, the date of diagnosis or first treatment does not equate to the date of disease, in particular because there are no specific tests to detect PD and because individuals are most commonly diagnosed years after symptom onset once the motor symptoms (e.g., freezing of gait) set in ^1,4–6^. Potential PD case misidentification cannot be excluded because PD cases were identified with either the date of LTI declaration, ODC declaration, or first treatment reimbursement. However, diagnostic misclassifications are unlikely to depend on the farming type^18^, and several sensitivity analyses were conducted to address this bias, which yielded consistent results (Supplementary material). Clinical information on PD FMs (e.g., severity scales, non-motor symptoms, disease phenotypes) as well as genetic information (from both causal and at-risk genetic variants) were not available. This prevented us from accounting for these factors in the analysis, which is a limitation. Indeed, given the complex nature of PD, which is characterized by a dynamic interplay between genes and the environment, some genetic variants (e.g., ABCB1) may increase susceptibility to pesticide exposures associated with PD^10,11,15,34,35,53,56,85^.

Because of the administrative nature of our data, some potential confounding factors of interest (e.g., cigarette smoking, genetic information, use of protective equipment) were not available^17,30,74^. This could represent a bias if their absence confounds or masks the genuine relationship between farming activities and PD. This is particularly true for cigarette smoking, which is a protective factor in PD^4,6,8,12,13,35^. Even though FMs have the lowest smoking prevalence in the French population, some of the differences observed may be associated with smoking because the prevalence of active smoking varies from one farming activity to another^86,87^. To address this bias, we conducted a sensitivity analysis, which yielded consistent results (Supplementary material). The possibility of residual confounding cannot be excluded because some farming activities are highly heterogeneous in nature, with, for instance, crop and fruit farming that involve a broad range of practices/tasks and pesticide usage depending on the type of crop (e.g., cereal, potatoes, barley, wheat) or fruit (e.g., walnut, apple). Residual confounding due to environmental exposure (e.g., air pollution in regions with a high density of industrial plants) should be limited because we adjusted for farm locations. While specific chemical, physical, or biological exposures were not available, farming activity serves as a relevant proxy for agricultural exposure that is easier to assess than specific pesticides or stressors^15,18–21,29^. To address this limitation and complement this study, job and crop exposure matrices could refine exposure estimates by enabling cumulative exposure indexing for pesticides, heavy metals, and organizational factors^88–92^. Undertaking such a task, which is beyond the scope of this study, would present major challenges, as it entails navigating various potential biases and limitations that must be carefully considered and addressed. The farming activity coding system used by Mutualité Sociale Agricole (MSA) is not an international classification, which may hinder the transcoding process. Some farming activities (e.g., crop farming) may be too broad (not descriptive enough) to allow for an accurate exposure assessment using crop or job exposure matrices. Finding relevant crop or job exposure matrices is also challenging due to the lack of a gold standard^93^. In addition, crop or job exposure matrices have also some limitations that may bias risk estimates, such as an heterogeneity in performance depending on the exposure and outcome of interest as well as the assumption of homogeneity within jobs/categories^88–90,93–95^. Ideally, the crop or job exposure matrix should define each farming activity as specifically as possible, with exposure estimates given for a minimal combination of the year, country, region, sex, work status (e.g., FMs or farm workers), and farming activity. However, to the best of our knowledge, such matrices do not exist, at least not in France. Because farming practices can differ from a country to another, as well as within countries, it is important to use crop and job exposure matrices that are comparable to the population studied. To our knowledge, no French job exposure matrix has been specifically designed for farming. Consequently, several crop or job exposure matrices would have to be used. However, each crop or job exposure matrix is created by potentially different experts, aims, and methodologies, which could make them not easily comparable and compatible with one another^88–90,93–95^. In France, there are several crop and job exposure matrices, in particular Matgéné^96^ and Pestimat^88^. For Matgéné^96^, exposure to pesticides is not provided, exposure estimates are not available for each sex and for each region, and exposure probabilities are provided as ranges. While French activity nomenclature (NAF) codes are available in both Matgéné^96^ and MSA data, NAF codes from MSA are, however, not reliable due to their declarative nature and non-evolving nature (i.e., rarely updated). Regarding Pestimat^88^, only exposure to pesticides is available, but not for the entire France and not for all types of crops^97^. Exposure estimates are not available for each sex and for each region. In addition, exposure estimates are only known for crop, vegetable, and fruit farming activities. However, pesticides, and in particular insecticides, are used in animal farming as well^19^. Moreover, the farming activities (e.g., crop farming) in MSA data are too broad compared to the information from Pestimat^88^ (e.g., potato farming), which would force us to consider that each crop farmer, regardless of the crops, is exposed to the same pesticides, which would introduce bias and exposure misclassification. Linking crop and job exposure matrices with MSA data is an issue that deserves to be explored in the future, as highlighted by a previous work^97^. Standardizing or developing new matrices tailored to French agriculture is therefore essential. Another perspective would be to investigate whether FMs engaged in various farming activities have a higher PD risk compared to a non-farming population.

Because occupational data was only available between 2002–2016 and because the average age at baseline was 47 years old, the exposure characterization only took into account the most recent half of the individuals’ careers. However, FMs have a relatively stable career because most FMs (90%) never changed their main activity between 2002-2016. Hence, the impact of this bias should be limited. The generalizability of our findings may be limited as farming practices and risk factors can differ between countries and populations (e.g., farm workers, FMs).

Our findings represent a preliminary work that suggests the potential involvement of occupational risk factors related to farming in PD onset and development. Our study could guide future research aiming to examine such factors by identifying vulnerable populations and potential research avenues. Future work should focus on farmers engaged in high-risk farming activities (i.e., pig farming, crop farming, cattle farming, mixed farming, viticulture, truck farming, and fruit arboriculture) to better characterize their exposome and potential association with PD because the combined exposure to multiple stressors (e.g., cocktail effect) may result in a synergistic adverse effect on PD risk, as alluded to by several studies in the literature. Confirmation of our findings in longitudinal studies and in other countries would also be valuable.

## Methods

### Data source

A secondary use of routinely collected data from Mutualité Sociale Agricole (MSA), the unique social security scheme of all French farmers, available to the TRACTOR (Tracking and monitoring occupational risks in agriculture) project was conducted^17,30,74,98–100^.

Insurance claim data routinely collected through the completion of mandatory forms by FMs on an annual basis from 2002 to 2016 was available. These claims provide information on sociodemographic (e.g., age, sex, family status), farm characteristics (e.g., farm surface), and farming activities. Farming activities are coded into 26 categories (e.g., pig farming, viticulture) by MSA following a national thesaurus defined by French laws^30^.

Electronic health/medical records from 2012–2016 were also available. These records provide information on declared chronic illnesses (LTIs), such as PD (LTI n°16), that grant full coverage of health care expenditures to FMs who hold them. Electronic health/medical records also pertain to ODCs, such as “*PD induced by pesticide exposures*” (ODC n°RA 58 in the agricultural scheme table). Since 2012, in France, PD can be considered a work-related disease in farmers under specific conditions, including a diagnosis confirmed by a neurologist and occupational exposure to pesticides for 10 years or more^7^. Data on drug reimbursements was also available. Each LTI and ODC is assigned an ICD-10 code (10th revision of the International Statistical Classification of Diseases and Related Health Problems), while each drug is assigned an ATC code (Anatomical Therapeutic Chemical classification system). January 1st, 2012, was defined as the baseline time point (i.e., time zero), and December 31st, 2016, as the follow-up end. The Kaplan-Meier reverse method was used to determine the median follow-up.

The data was analyzed from September 2023 to September 2024. The variables used for this study were complete. Strengthening the Reporting of Observational Studies in Epidemiology (STROBE) was used as reporting guidelines (Supplementary Table 6).

### Ethics approval

This study was conducted in accordance with the Declaration of Helsinki, and ethics approval was obtained from the French independent administrative authority responsible for safeguarding privacy and personal data (CNIL) (authorization number: MMS/SBM/AE171001). The need for informed consent was waived by CNIL (i.e., ethics committee) for the TRACTOR project because data analyses were only descriptive and results were reported at a large collective scale (i.e., farming activity level), because data were pseudomyzed, and because measures were undertaken to prevent the risk of reidentification of individuals.

### Study population and outcome

All FMs who performed at least one of the 26 activities once (1 yearly declaration to MSA) between 2002 and 2016 were included. The farming activity was considered a proxy for occupational exposure (agricultural activity exposomes), as previously done in other works^17–21^. The FMs’ degree of involvement in the daily tasks was unknown (not recorded by MSA). The duration of exposure for each activity was determined by calculating the number of years in which a FM engaged in the activity based on the yearly declarations made to MSA during the period from 2002 to 2016.

PD cases were identified using ICD-10 codes for FMs declared with PD through the LTI and ODC insurance declaration schemes, as well as with ATC codes for PD drugs given to FMs (with or without LTI or ODC)^20–22,101^. FMs were considered to have PD if they had at least one LTI declaration for PD (ICD-10 code G20 or F02), one ODC declaration for PD, or one reimbursement of any drugs solely used to treat PD (i.e., all antiparkinsonian agents, with the exception of pramipexole, rotigotine, amantadine, and lisuride) (Supplementary Table 7). In addition, FMs only on anticholinergics (trihexyphenidyl, biperiden, and tropatepine) and neuroleptics (drug-induced parkinsonism) were not considered as PD cases.

### Statistical analysis

All statistical analyses were performed with R software 4.3.1® (R Core Team, Vienna, Austria) for Windows 10©. When the number of exposed PD cases was ≥3, Cox proportional hazards models and associated hazard ratios (HRs) were used to investigate whether specific farming activities exhibited a higher risk of PD than others, both overall and by sex category (i.e., one separate model for each sex). Because we did not have access to the general population nor to other occupational sectors not related to agriculture, a separate model was created for each of the 26 farming activities, comparing FMs who had not previously engaged in a given farming activity from 2002 to 2016 with those who had prior experience (leave-one-group-out approach).

For each model, the dependent variables were the timescale (continuous) and the PD diagnosis (two categories: yes or no). The time to the oldest PD insurance declaration (LTI or ODC) or PD drug reimbursement was used as the underlying timescale. In addition, the farming activity was parameterized as a time-dependent variable to account for potential immortal time bias.

Overall analyses were adjusted for sex, with interaction tests conducted to assess potential sex differences. All analyses were adjusted for age, first year of the farm’s establishment, median farm surface, number of associates, unemployment status, total number of farms, family status, partner work status, farm location, number of comorbidities, and performing a secondary farming activity (Supplementary Table 8). We ensured that the included variables were non-collinear (variance inflation factor ≤ 2.5). A covariate*time interaction was added to the model when the assumption of a proportional hazard rate, assessed by the independence of scaled Schoenfeld’s residuals and time, was not met. Multiple testing was accounted for using the Benjamini-Hochberg approach.

Seventeen sensitivity analyses (SAs) were undertaken to test hypotheses and address potential sources of bias (Supplementary Table 8). For example, PD cases diagnosed in 2012 were excluded to increase the likelihood that identified PD cases were incident cases (SA1). In another SA (SA2), the PD case identification was based solely on ODC declarations. Please refer to the Supplementary material for more details.

## Supplementary information

Supplementary material

Supplementary Tables 4 and 5

## Data Availability

The data that supports the findings of this study is not publicly available. A reasonable request to the Mutualité Sociale Agricole (MSA) can be made, but restrictions apply to the availability of these data due to both the individual and medical nature of the data, which requires approval from both the MSA and the French independent administrative authority protecting privacy and personal data (CNIL). Further information is available from the corresponding author upon request.

## Code availability

The underlying code for this study is not publicly available for proprietary reasons but can be made available upon reasonable request.
