# Supplementary material for: Investigating Parkinson’s disease risk across farming activities using data mining and large-scale administrative health data
Source: NPJ Parkinsons Dis. 2025 Jan 8;11:13. doi: 10.1038/s41531-024-00864-2 (PMC11711245; doi:10.1038/s41531-024-00864-2)
Supplement: Supplementary file 1 — Supplementary material [file 41531_2024_864_MOESM1_ESM.docx]

**Supplementary material**

**Title**

Investigating Parkinson’s disease risk across farming activities using data mining and large-scale administrative health data

**Authors**

Pascal Petit, François Berger, Vincent Bonneterre, Nicolas Vuillerme

**Table of contents**

[Supplementary Table 1: Number of farm managers and identified PD cases for each analysis conducted 3](#_Toc182386259)

[Supplementary Table 2: Incidence rate per analysis 4](#_Toc182386260)

[Supplementary Table 3: Number of farm managers with a LTI, ODC or ATC code of interest 5](#_Toc182386261)

[Supplementary Table 4: Farming activities and risks of PD, TRACTOR project, France, 2012-2016. Comparison of the main analysis with all sensitivity analyses for all sex categories. 6](#_Toc182386262)

[Supplementary Table 5: Comparison of results from the interaction tests regarding the sex between the main analysis and all sensitivity analyses. 6](#_Toc182386263)

[Supplementary Table 6: STROBE Statement Checklist 7](#_Toc182386264)

[Supplementary Table 7: ICD-10 and ATC codes used for identifying PD cases among farm managers depending on the analysis 8](#_Toc182386265)

[Supplementary Table 8: List of covariates considered in the statistical analyses 9](#_Toc182386266)

[Sensitivity analysis 10](#_Toc182386267)

[*Sensitivity analysis 1 - excluding PD cases diagnosed in 2012* 10](#_Toc182386268)

[Supplementary Figure 1: Farming activities and risks of Parkinson’s disease, TRACTOR project, 2012-2016 – sensitivity analysis 1 11](#_Toc182386269)

[*Sensitivity analysis 2 – PD identification using only ODC declaration data* 12](#_Toc182386270)

[Supplementary Figure 2: Farming activities and risks of Parkinson’s disease, TRACTOR project, 2012-2016 – sensitivity analysis 2 13](#_Toc182386271)

[*Sensitivity analyses 3 to 5 – age restrictions* 14](#_Toc182386272)

[Supplementary Figure 3: Farming activities and risks of Parkinson’s disease, TRACTOR project, 2012-2016 – sensitivity analysis 3 15](#_Toc182386273)

[Supplementary Figure 4: Farming activities and risks of Parkinson’s disease, TRACTOR project, 2012-2016 – sensitivity analysis 4 16](#_Toc182386274)

[Supplementary Figure 5: Farming activities and risks of Parkinson’s disease, TRACTOR project, 2012-2016 – sensitivity analysis 5 17](#_Toc182386275)

[*Sensitivity analyses 6 to 14 – addressing potential case misclassification* 18](#_Toc182386276)

[Supplementary Figure 6: Farming activities and risks of Parkinson’s disease, TRACTOR project, 2012-2016 – sensitivity analysis 6 21](#_Toc182386277)

[Supplementary Figure 7: Farming activities and risks of Parkinson’s disease, TRACTOR project, 2012-2016 – sensitivity analysis 7 22](#_Toc182386278)

[Supplementary Figure 8: Farming activities and risks of Parkinson’s disease, TRACTOR project, 2012-2016 – sensitivity analysis 8 23](#_Toc182386279)

[Supplementary Figure 9: Farming activities and risks of Parkinson’s disease, TRACTOR project, 2012-2016 – sensitivity analysis 9 24](#_Toc182386280)

[Supplementary Figure 10: Farming activities and risks of Parkinson’s disease, TRACTOR project, 2012-2016 – sensitivity analysis 10 25](#_Toc182386281)

[Supplementary Figure 11: Farming activities and risks of Parkinson’s disease, TRACTOR project, 2012-2016 – sensitivity analysis 11 26](#_Toc182386282)

[Supplementary Figure 12: Farming activities and risks of Parkinson’s disease, TRACTOR project, 2012-2016 – sensitivity analysis 12 27](#_Toc182386283)

[Supplementary Figure 13: Farming activities and risks of Parkinson’s disease, TRACTOR project, 2012-2016 – sensitivity analysis 13 28](#_Toc182386284)

[Supplementary Figure 14: Farming activities and risks of Parkinson’s disease, TRACTOR project, 2012-2016 – sensitivity analysis 14 29](#_Toc182386285)

[*Sensitivity analysis 15 – using only one farming activity as reference* 30](#_Toc182386286)

[Supplementary Figure 15: Farming activities and risks of Parkinson’s disease, TRACTOR project, 2012-2016 – sensitivity analysis 15 31](#_Toc182386287)

[*Sensitivity analysis 16 – adjusting for smoking* 32](#_Toc182386288)

[Supplementary Figure 16: Farming activities and risks of Parkinson’s disease, TRACTOR project, 2012-2016 – sensitivity analysis 16 33](#_Toc182386289)

[*Sensitivity analysis 17 – control of other diseases not related to farming exposure* 34](#_Toc182386290)

[Supplementary Figure 17: Farming activities and risks of Parkinson’s disease, TRACTOR project, 2012-2016 – sensitivity analysis 17 36](#_Toc182386291)

[References 37](#_Toc182386292)

# **Supplementary Table 1**: Number of farm managers and identified PD cases for each analysis conducted

| **Analysis** | **FMs without PD** No. (%) | **FMs with PD** No. (%) | **Total number**  **of FMs** |
| --- | --- | --- | --- |
| Main analysis | 1079716 (99.2%) | 8845 (0.81%) | 1088561 |
| Sensitivity analysis 1 - excluding PD cases diagnosed in 2012 | 1079716 (99.4%) | 5910 (0.54%) | 1085626 |
| Sensitivity analysis 2 - PD identification only using ODC declaration data | 1088244 (99.9%) | 317 (0.03%) | 1088561 |
| Sensitivity analysis 3 - only including FMs ≥ 40 years | 715050 (98.9%) | 7893 (1.09%) | 722943 |
| Sensitivity analysis 4 - only including FMs ≥ 50 years | 446334 (98.6%) | 6388 (1.41%) | 452722 |
| Sensitivity analysis 5 - only including FMs ≥ 60 years | 165468 (98.1%) | 3206 (1.90%) | 168674 |
| Sensitivity analysis 6 - PD identification only using ODC and LTI declaration data | 1085439 (99.7%) | 3122 (0.29%) | 1088561 |
| Sensitivity analysis 7 - PD identification only using drug reimbursement data | 1080343 (99.2%) | 8218 (0.76%) | 1088561 |
| Sensitivity analysis 8 - excluding FMs with ADRD (LTI declaration) | 1080113 (99.2%) | 8448 (0.78%) | 1088561 |
| Sensitivity analysis 9 - PD identification only using LTI declaration data (G20, G21) | 1085449 (99.7%) | 3112 (0.29%) | 1088561 |
| Sensitivity analysis 10 - PD identification only using LTI declaration data (F02, G20, G21) | 1085534 (99.7%) | 3027 (0.28%) | 1088561 |
| Sensitivity analysis 11 - considering drug reimbursement not solely used for PD | 1078438 (99.1%) | 10123 (0.93%) | 1088561 |
| Sensitivity analysis 12 - PD identification using LTI, ODC or ≥ 2 drug reimbursements | 1081419 (99.3%) | 7142 (0.66%) | 1088561 |
| Sensitivity analysis 13 - PD identification using LTI, ODC or ≥ 3 drug reimbursements | 1081951 (99.4%) | 6610 (0.61%) | 1088561 |
| Sensitivity analysis 14 - PD identification using LTI, ODC or ≥ 6 drug reimbursements | 1082990 (99.5%) | 5571 (0.51%) | 1088561 |
| Sensitivity analysis 15 - using only one farming activity as reference | 1079716 (99.2%) | 8845 (0.81%) | 1088561 |
| Sensitivity analysis 16 - adjusting on smoking | 1079716 (99.2%) | 8845 (0.81%) | 1088561 |
| Sensitivity analysis 17 - control of other diseases not related to farming exposure | 530961 (98.4%) | 8845 (1.64%) | 539806 |

Abbreviations: ADRD: Alzheimer’s disease and other related dementias, F02: ICD-10 code for dementia in other diseases classified elsewhere, FM: farm manager, G20: ICD-10 code for Parkinson’s disease, G21: ICD-10 code for secondary parkinsonism, ICD-10: 10^th^ revision of the International Statistical Classification of Diseases and Related Health Problems, LTI: long-term illness, No: number of, ODCs: occupational diseases covered under workers’ compensation statutes, PD: Parkinson’s disease.

# **Supplementary Table 2**: Incidence rate per analysis

| **Analysis** | **Both sexes** | | **Male** | | **Female** | |
| --- | --- | --- | --- | --- | --- | --- |
|  | **n** | **IR [95%CI]^a^** | **n** | **IR [95%CI]^a^** | **n** | **IR [95%CI]^a^** |
| Main analysis | 8845 | 0.28 [0.27-0.29] | 5500 | 0.40 [0.38-0.41] | 3345 | 0.24 [0.23-0.25] |
| Sensitivity analysis 1 - excluding PD cases diagnosed in 2012 | 5910 | 0.19 [0.18-0.20] | 3713 | 0.26 [0.25-0.27] | 2197 | 0.16 [0.15-0.17] |
| Sensitivity analysis 2 - PD identification only using ODC declaration data | 317 | 0.01 [0.009-0.02] | 285 | 0.01 [0.009-0.02] | 32 | 0.004 [0.003-0.005] |
| Sensitivity analysis 3 - only including FMs ≥ 40 years | 7893 | 0.49 [0.48-0.50] | 4718 | 0.59 [0.57-0.61] | 3175 | 0.44 [0.43-0.46] |
| Sensitivity analysis 4 - only including FMs ≥ 50 years | 6388 | 1.04 [1.02-1.07] | 3575 | 1.09 [1.06-1.13] | 2813 | 0.98 [0.95-1.02] |
| Sensitivity analysis 5 - only including FMs ≥ 60 years | 3206 | 1.72 [1.66-1.78] | 1544 | 1.62 [1.54-1.71] | 1662 | 1.82 [1.73-1.91] |
| Sensitivity analysis 6 - PD identification only using ODC and LTI declaration data | 3122 | 0.10 [0.08-0.11] | 2083 | 0.12 [0.11-0.13] | 1039 | 0.09 [0.08-0.10] |
| Sensitivity analysis 7 - PD identification only using drug reimbursement data | 8218 | 0.26 [0.25-0.27] | 5104 | 0.37 [0.36-0.38] | 3114 | 0.22 [0.21-0.23] |
| Sensitivity analysis 8 - excluding FMs with ADRD (LTI declaration) | 8448 | 0.27 [0.26-0.28] | 5302 | 0.37 [0.36-0.39] | 3146 | 0.23 [0.22-0.24] |
| Sensitivity analysis 9 - PD identification only using LTI declaration data (G20, G21) | 3112 | 0.11 [0.08-0.12] | 2077 | 0.12 [0.11-0.13] | 1035 | 0.09 [0.08-0.10] |
| Sensitivity analysis 10 - PD identification only using LTI declaration data (F02, G20, G21) | 3027 | 0.10 [0.09-0.12] | 1994 | 0.12 [0.11-0.13] | 1033 | 0.09 [0.08-0.10] |
| Sensitivity analysis 11 - considering drug reimbursement not solely used for PD | 10123 | 0.32 [0.31-0.33] | 6206 | 0.47 [0.45-0.48] | 3917 | 0.27 [0.26-0.28] |
| Sensitivity analysis 12 - PD identification using LTI, ODC or ≥ 2 drug reimbursements | 7142 | 0.23 [0.22-0.24] | 4481 | 0.32 [0.30-0.33] | 2661 | 0.20 [0.19-0.21] |
| Sensitivity analysis 13 - PD identification using LTI, ODC or ≥ 3 drug reimbursements | 6610 | 0.21 [0.20-0.22] | 4163 | 0.29 [0.28-0.30] | 2447 | 0.18 [0.17-0.19] |
| Sensitivity analysis 14 - PD identification using LTI, ODC or ≥ 6 drug reimbursements | 5571 | 0.18 [0.17-0.19] | 3641 | 0.25 [0.24-0.26] | 2130 | 0.16 [0.15-0.17] |
| Sensitivity analysis 15 - using only one farming activity as reference | 8845 | 0.28 [0.27-0.29] | 5500 | 0.40 [0.38-0.41] | 3345 | 0.24 [0.23-0.25] |
| Sensitivity analysis 16 - adjusting on smoking | 8845 | 0.28 [0.27-0.29] | 5500 | 0.40 [0.38-0.41] | 3345 | 0.24 [0.23-0.25] |
| Sensitivity analysis 17 - control of other diseases not related to farming exposure | 8845 | 0.81 [0.79-0.83] | 5500 | 1.00 [0.96-1.03] | 3345 | 0.73 [0.71-0.75] |

Abbreviations: ADRD: Alzheimer’s disease and other related dementias, F02: ICD-10 code for dementia in other diseases classified elsewhere, FM: farm manager, G20: ICD-10 code for Parkinson’s disease, G21: ICD-10 code for secondary parkinsonism, ICD-10: 10^th^ revision of the International Statistical Classification of Diseases and Related Health Problems, IR: incidence rate, LTI: long-term illness, ODCs: occupational diseases covered under workers’ compensation statutes, PD: Parkinson’s disease, PR: prevalence.

^a^ incidence rates (IRs) are expressed as cases per 1000 persons-years.

# **Supplementary Table 3**: Number of farm managers with a LTI, ODC or ATC code of interest

1. Number of PD farm managers with a PD drug reimbursement based on the drug name

| **ATC code** | **Drug name** | **Indication*** | **Number of FMs**  No. (%) |
| --- | --- | --- | --- |
| N04 | Anti-parkinson drugs | PD and other disorders (cf. below) | 10123 (0.93) |
| N04 | Anti-parkinson drugs solely used for PD | PD only (cf. below) | 8845 (0.81) |
| N04BA02 | Levodopa and decarboxylase inhibitor | PD only | 2698 (0.25) |
| N04BC08 | Piribedil | PD only | 2286 (0.21) |
| N04BC05 | Pramipexole | PD, restless legs syndrome | 2056 (0.19) |
| N04BC04 | Ropinirole | PD only | 1468 (0.13) |
| N04BD02 | Rasagiline | PD only | 1300 (0.12) |
| N04AA01 | Trihexyphenidyl | PD, parkinsonism | 1125 (0.10) |
| N04AA12 | Tropatepine | PD, parkinsonism | 1009 (0.09) |
| N04BA03 | Levodopa, decarboxylase inhibitor and COMT inhibitor | PD only | 873 (0.08) |
| N04BC09 | Rotigotine | PD, restless legs syndrome | 416 (0.04) |
| N04BB01 | Amantadine | PD, influenza A, postherpetic neuralgia | 253 (0.02) |
| N04AA02 | Biperiden | PD, parkinsonism | 168 (0.02) |
| N04BX02 | Entacapone | PD only | 110 (0.01) |
| N04BC07 | Apomorphine | PD only | 102 (0.01) |
| N04BD01 | Selegiline | PD only | 32 (2.9e-03) |
| N04BC01 | Bromocriptine | PD only | 19 (1.7e-03) |
| N04BX01 | Tolcapone | PD only | 9 (8.3e-04) |
| N02CA07 | Lisuride | PD, hyperprolactinaemia disorders | 0 |
| N04BA01 | Levodopa | PD only | 0 |
| N04BC02 | Pergolide | PD only | 0 |

Abbreviations: COMT: catechol-O-methyltransferase, FM: farm manager, No: number of, PD: Parkinson’s disease.

*According to the Vidal (<https://www.vidal.fr/>), Thesorimed (<https://theso.prod-un.thesorimed.org/monographie>) and BCB databases (<https://www.bcb.fr/v2/app/recherche.jsp>).

1. Number of PD farm managers with a LTI or ODC declaration based on the ICD-10 code

| **ICD-10 code** | **Definition** | **Number of FMs**  No. (%) |
| --- | --- | --- |
| G20 | Parkinson’s disease | 3034 (0.28) |
| G21 | Secondary parkinsonism | 98 (9.0e-03) |
| F02 | Dementia in other diseases classified elsewhere | 12 (1.1e-03) |

Abbreviations: ICD-10: 10^th^ revision of the International Statistical Classification of Diseases and Related Health Problems, FM: farm manager, No: number of, ODCs: occupational diseases covered under workers’ compensation statutes, PD: Parkinson’s disease.

# **Supplementary Table 4**: Farming activities and risks of PD, TRACTOR project, France, 2012-2016. Comparison of the main analysis with all sensitivity analyses for all sex categories.

Please refer to the MS Excel file entitled *Supplementary Tables 4 and 5*, in the tab named “*Supplementary Table 4*”.

# **Supplementary Table 5**: Comparison of results from the interaction tests regarding the sex between the main analysis and all sensitivity analyses.

Please refer to the MS Excel file entitled *Supplementary Tables 4 and 5*, in the tab named “*Supplementary Table 5*”.

# **Supplementary Table 6**: STROBE Statement Checklist

|  | Item  No | Recommendation | Page |
| --- | --- | --- | --- |
| **Title and abstract** | 1 | (*a*) Indicate the study’s design with a commonly used term in the title or the abstract | 1, 2 |
|  |  | (*b*) Provide in the abstract an informative and balanced summary of what was done and what was found | 2 |
| Introduction | | |  |
| Background/rationale | 2 | Explain the scientific background and rationale for the investigation being reported | 3 |
| Objectives | 3 | State specific objectives, including any prespecified hypotheses | 3, 4 |
| Methods | | |  |
| Study design | 4 | Present key elements of study design early in the paper | 14, 15 |
| Setting | 5 | Describe the setting, locations, and relevant dates, including periods of recruitment, exposure, follow-up, and data collection | 14, 15 |
| Participants | 6 | (*a*) Give the eligibility criteria, and the sources and methods of selection of participants. Describe methods of follow-up | 14, 15 |
|  |  | (*b*) For matched studies, give matching criteria and number of exposed and unexposed | DNA |
| Variables | 7 | Clearly define all outcomes, exposures, predictors, potential confounders, and effect modifiers. Give diagnostic criteria, if applicable | 15-17, suppl |
| Data sources/ measurement | 8* | For each variable of interest, give sources of data and details of methods of assessment (measurement). Describe comparability of assessment methods if there is more than one group | 15-17, suppl |
| Bias | 9 | Describe any efforts to address potential sources of bias | 16,17, suppl |
| Study size | 10 | Explain how the study size was arrived at | 14-16 |
| Quantitative variables | 11 | Explain how quantitative variables were handled in the analyses. If applicable, describe which groupings were chosen and why | 15-17, suppl |
| Statistical methods | 12 | (*a*) Describe all statistical methods, including those used to control for confounding | 15-17, suppl |
|  |  | (*b*) Describe any methods used to examine subgroups and interactions | 15-17, suppl |
|  |  | (*c*) Explain how missing data were addressed | 15-17, suppl |
|  |  | (*d*) If applicable, explain how loss to follow-up was addressed | DNA |
|  |  | (*e*) Describe any sensitivity analyses | 16, 17, suppl |
| Results | | |  |
| Participants | 13* | (a) Report numbers of individuals at each stage of study-e.g., numbers potentially eligible, examined for eligibility, confirmed eligible, included in the study, and analyzed | 4 |
|  |  | (b) Give reasons for non-participation at each stage | DNA |
|  |  | (c) Consider use of a flow diagram | DNA |
| Descriptive data | 14* | (a) Give characteristics of study participants (e.g., demographic, clinical, social) and information on exposures and potential confounders | 4, Table 1 |
|  |  | (b) Indicate number of participants with missing data for each variable of interest | DNA |
|  |  | (c) Summarize follow-up time (e.g., average and total amount) | 4, suppl |
| Outcome data | 15* | Report numbers of outcome events or summary measures over time | 4, suppl |
| Main results | 16 | (*a*) Give unadjusted estimates and, if applicable, confounder-adjusted estimates and their precision (e.g., 95% confidence interval). Make clear which confounders were adjusted for and why they were included | 4-7, suppl |
|  |  | (*b*) Report category boundaries when continuous variables were categorized | DNA |
|  |  | (*c*) If relevant, consider translating estimates of relative risk into absolute risk for a meaningful time period | DNA |
| Other analyses | 17 | Report other analyses done-e.g., analyses of subgroups and interactions, and sensitivity analyses | 5-7, suppl |
| Discussion | | |  |
| Key results | 18 | Summarize key results with reference to study objectives | 8 |
| Limitations | 19 | Discuss limitations of the study, taking into account sources of potential bias or imprecision. Discuss both direction and magnitude of any potential bias | 8, 12-14 |
| Interpretation | 20 | Give a cautious overall interpretation of results considering objectives, limitations, multiplicity of analyses, results from similar studies, and other relevant evidence | 8, 12-14 |
| Generalizability | 21 | Discuss the generalizability (external validity) of the study results | 14 |
| Other information | | |  |
| Funding | 22 | Give the source of funding and the role of the funders for the present study and, if applicable, for the original study on which the present article is based | 18 |

*Give information separately for exposed and unexposed groups.

DNA: does not applied, suppl: supplementary material.

# **Supplementary Table 7**: ICD-10 and ATC codes used for identifying PD cases among farm managers depending on the analysis

| **Data origin** | **Classification**  **system** | **Code** | **Definition** | **Drug**  **Indication^§^** | **Analysis** |
| --- | --- | --- | --- | --- | --- |
| Long-term illness scheme/declaration *(requirement to be considered as a PD case: at least one declaration of any of these three ICD-10 codes)* | ICD-10 | F02 | Dementia in other diseases classified elsewhere | / | MA, SA1, SA3-6, SA8, SA10-17 |
|  | ICD-10 | G20 | Parkinson’s disease | / | MA, SA1, SA3-6, SA8-17 |
|  | ICD-10 | G21 | Secondary parkinsonism | / | SA6, SA9-11 |
| Occupational disease scheme  *(requirement to be considered as a PD case: at least one declaration for PD induced by pesticides)* | ODC | RA 58 | PD induced by pesticide exposure | / | MA, SA1-6, SA8, SA11-17 |
| Drug reimbursement for PD  *(requirement to be considered as a PD case: at least one reimbursement of any of these ATC codes)* | ATC | N04BA01 | Levodopa | PD only | MA, SA1, SA3-5, SA7-8, SA11-17 |
|  | ATC | N04BA02 | Levodopa and decarboxylase inhibitor | PD only | MA, SA1, SA3-5, SA7-8, SA11-17 |
|  | ATC | N04BA03 | Levodopa, decarboxylase inhibitor and COMT inhibitor | PD only | MA, SA1, SA3-5, SA7-8, SA11-17 |
|  | ATC | N04BD01 | Selegiline | PD only | MA, SA1, SA3-5, SA7-8, SA11-17 |
|  | ATC | N04BD02 | Rasagiline | PD only | MA, SA1, SA3-5, SA7-8, SA11-17 |
|  | ATC | N04BX01 | Tolcapone | PD only | MA, SA1, SA3-5, SA7-8, SA11-17 |
|  | ATC | N04BX02 | Entacapone | PD only | MA, SA1, SA3-5, SA7-8, SA11-17 |
|  | ATC | N04BC01 | Bromocriptine | PD only | MA, SA1, SA3-5, SA7-8, SA11-17 |
|  | ATC | N04BC04 | Ropinirole | PD only | MA, SA1, SA3-5, SA7-8, SA11-17 |
|  | ATC | N04BC07 | Apomorphine | PD only | MA, SA1, SA3-5, SA7-8, SA11-17 |
|  | ATC | N04BC08 | Piribedil | PD only | MA, SA1, SA3-5, SA7-8, SA11-17 |
|  | ATC | N04AA01 | Trihexyphenidyl* | PD, parkinsonism | MA, SA1, SA3-5, SA7-8, SA11-17 |
|  | ATC | N04AA02 | Biperiden* | PD, parkinsonism | MA, SA1, SA3-5, SA7-8, SA11-17 |
|  | ATC | N04AA12 | Tropatepine* | PD, parkinsonism | MA, SA1, SA3-5, SA7-8, SA11-17 |
|  | ATC | N02CA07 | Lisuride | PD, hyperprolactinemia disorders | SA11 |
|  | ATC | N04BB01 | Amantadine | PD, influenza A, postherpetic neuralgia | SA11 |
|  | ATC | N04BC05 | Pramipexole | PD, Restless Legs Syndrome | SA11 |
|  | ATC | N04BC09 | Rotigotine | PD, Restless Legs Syndrome | SA11 |

*Abbreviations*: ATC: Anatomical Therapeutic Chemical classification system, COMT: catechol-O-methyltransferase, ICD-10: 10^th^ revision of the International Statistical Classification of Diseases and Related Health Problems, MA: main analysis, ODCs: occupational diseases covered under workers’ compensation statutes, PD: Parkinson’s disease, SA: sensitivity analysis.

* Farm managers only on anticholinergics and neuroleptics (drug-induced parkinsonism) were not considered.

^§^  According to the Vidal (<https://www.vidal.fr/>), Thesorimed (<https://theso.prod-un.thesorimed.org/monographie>) and BCB databases (<https://www.bcb.fr/v2/app/recherche.jsp>).

# **Supplementary Table 8**: List of covariates considered in the statistical analyses

| **Dependent variable** | **Modality** |
| --- | --- |
| PD diagnosis (LTI declaration or drug reimbursement) | 2 categories: yes or no |
| Time to first PD insurance declaration, or drug reimbursement | continuous |
|  |  |
| **Independent variables** |  |
| Activity | 2 categories: yes or no |
| Sex^*^ | 2 categories: female or male |
| Age | continuous |
| First year of the farm’s establishment, years | continuous |
| Median yearly farm surface, hectares | continuous |
| Median yearly insurance premium, euros | continuous |
| Number of associates | continuous |
| Unemployment status | 2 categories: never unemployed or had been unemployed at least once over the period 2002-2016 |
| Number of farms | 2 categories: 1 or > 1 |
| Family status | 2 categories: single or as a couple |
| Partner work status | 2 categories: perform or do not perform task to help farm manager |
| Having a secondary farming activity^†^ | 2 categories: yes or no |
| Number of pre-existing medical comorbidities | continuous |
| Farm location | 96 categories: 96 metropolitan French administrative geographical areas (departments) |

*Abbreviations*: LTI: long-term illness, PD: Parkinson’s disease.

Categorical variables with more than two categories were converted to binary variables by applying the one-hot encoding approach, with the exclusion of one category to prevent multicollinearity. For each binary variable (e.g., activity), the reference was set as the ‘no’ or ‘0’ modality.

*The analysis was adjusted on sex only for “both sexes”; otherwise, sex was used for subgroup analyses.

† A secondary farming activity is defined as a farming activity (e.g., grassland farming) that a FM can perform in addition to its main activity (e.g., ovine farming). The nature of the secondary activity is, however, unknown.

# **Sensitivity analysis**

Thirteen sensitivity analyses (SAs) were undertaken to test hypotheses and address potential sources of bias.

## *Sensitivity analysis 1 - excluding PD cases diagnosed in 2012*

To increase the likelihood that identified PD cases were incident cases, a sensitivity analysis was conducted by excluding PD cases diagnosed in 2012. Similarly to the main analysis, for this sensitivity analysis, FMs were considered to have PD if they had at least one LTI declaration for PD (ICD-10 code G20 or F02), one ODC declaration for PD, or one reimbursement of any drugs solely used to treat PD (i.e., all antiparkinsonian agents, with the exception of pramipexole, rotigotine, amantadine, and lisuride) (Supplementary Table 7). However, FMs only on anticholinergics (trihexyphenidyl, biperiden, and tropatepine) and neuroleptics (drug-induced parkinsonism) were not considered PD cases.

The highest-risk group included FMs engaged in crop farming, mixed cattle farming, dairy farming, truck farming, pig farming, viticulture, unspecified and mixed farming (Supplementary Table 4, Supplementary Figure 1). A positive trend was also observed for ovine and caprine farming. By contrast, the lowest-risk group included FMs engaged in gardening, landscaping and reforestation companies, small animal farming, training, dressage and riding clubs, and stud farming. No farming activities exhibit a sex difference.


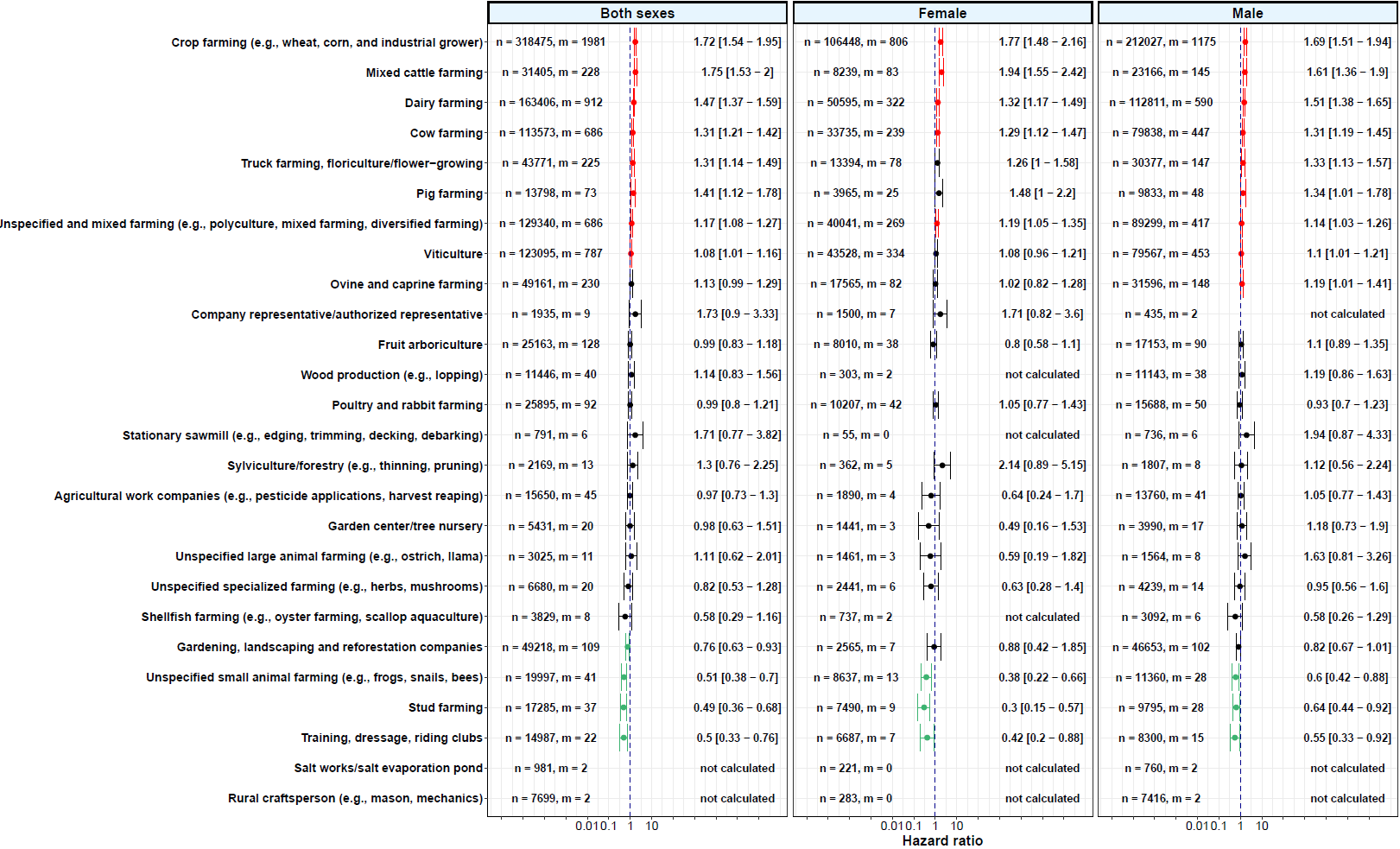


# **Supplementary Figure 1**: Farming activities and risks of Parkinson’s disease, TRACTOR project, 2012-2016 – sensitivity analysis 1

Multivariable Cox regression models for Parkinson’s disease according to each agricultural activity (y-axis) are displayed when the number of exposed cases was sufficient (m ≥ 3). The hazard ratio is represented by a point (x-axis), while error bars represent the 95% confidence interval. The red error bars refer to a higher risk of Parkinson’s disease while the green error bars represent a lower risk of Parkinson’s disease. The black error bars indicate situations where there is no difference in risk of Parkinson’s disease among the farm managers engaged in the considered activity compared to the population of farm managers not performing the considered activity. All analyses were adjusted for sex (for “both sexes” only), age, first year of the farm’s establishment, median farm surface, number of associates, unemployment status, total number of farms, family status, partner work status, farm location, number of comorbidities, and performing a secondary farming activity. n, number of exposed farm managers; m, number of exposed Parkinson’s disease cases.

## *Sensitivity analysis 2 – PD identification using only ODC declaration data*

In this sensitivity analysis, the PD case identification was based solely on ODC declarations for PD induced by pesticide exposures, knowing that compensation for PD as an occupational disease among long-term pesticide users, with causality presumption, only started in May 2012 in France^1^. The highest-risk group included FMs engaged in mixed cattle farming, pig farming, viticulture, and unspecified and mixed farming (Supplementary Table 4, Supplementary Figure 2). By contrast, no activity had reduced HRs, probably because of the small number of PD cases (317 in total) identified for this analysis. Viticulture was the only farming activity that exhibited a sex difference, with a PD risk around two times higher in females than males.


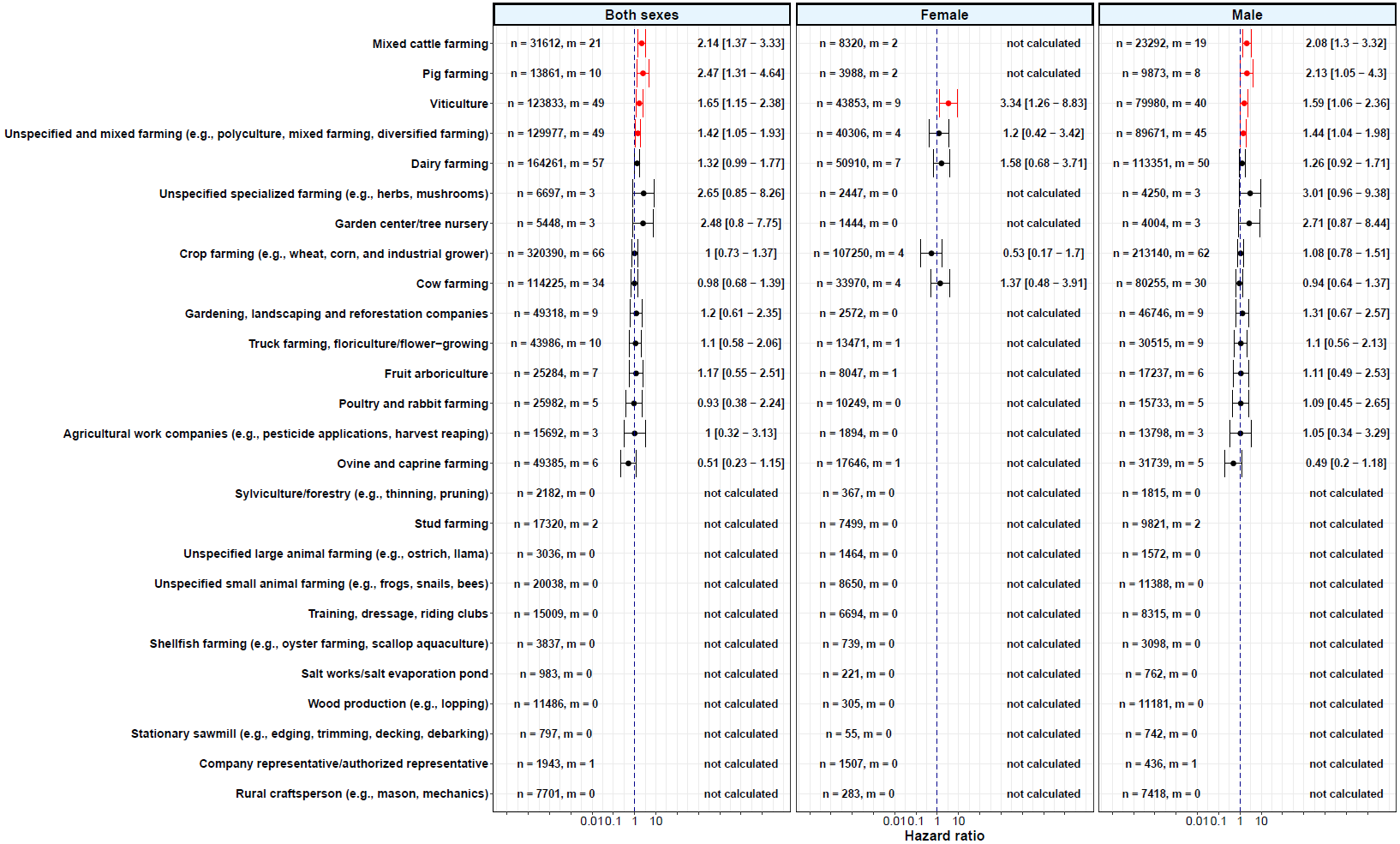


# **Supplementary Figure 2**: Farming activities and risks of Parkinson’s disease, TRACTOR project, 2012-2016 – sensitivity analysis 2

Multivariable Cox regression models for Parkinson’s disease according to each agricultural activity (y-axis) are displayed when the number of exposed cases was sufficient (m ≥ 3). The hazard ratio is represented by a point (x-axis), while error bars represent the 95% confidence interval. The red error bars refer to a higher risk of Parkinson’s disease while the green error bars represent a lower risk of Parkinson’s disease. The black error bars indicate situations where there is no difference in risk of Parkinson’s disease among the farm managers engaged in the considered activity compared to the population of farm managers not performing the considered activity. All analyses were adjusted for sex (for “both sexes” only), age, first year of the farm’s establishment, median farm surface, number of associates, unemployment status, total number of farms, family status, partner work status, farm location, number of comorbidities, and performing a secondary farming activity. n, number of exposed farm managers; m, number of exposed Parkinson’s disease cases.

## *Sensitivity analyses 3 to 5 – age restrictions*

Because PD is not common before age 40^1-3^, one SA was restricted to FMs who were 40 years and older (SA3), another one to FMs who were 50 years and older (SA4), and a third to FMs who were60 years and older (SA5), respectively. Similarly to the main analysis, for these sensitivity analyses, FMs were considered to have PD if they had at least one LTI declaration for PD (ICD-10 code G20 or F02), one ODC declaration for PD, or one reimbursement of any drugs solely used to treat PD (i.e., all antiparkinsonian agents, with the exception of pramipexole, rotigotine, amantadine, and lisuride) (Supplementary Table 7). However, FMs only on anticholinergics (trihexyphenidyl, biperiden, and tropatepine) and neuroleptics (drug-induced parkinsonism) were not considered PD cases.

Regarding the sensitivity analysis restricted to FMs ≥ 40 years (SA3), the highest-risk group included FMs engaged in mixed cattle farming, pig farming, dairy farming, cow farming, crop farming, unspecified and mixed farming, truck farming, and fruit arboriculture (Supplementary Table 4, Supplementary Figure 3). A positive trend was also observed for viticulture and ovine and caprine farming. By contrast, the lowest-risk group included FMs engaged in gardening, landscaping and reforestation companies, small animal farming, training, dressage and riding clubs, stud farming, shellfish farming and rural craftsperson. Dairy farming and stud farming were the only activities exhibiting a sex difference, with male FMs having a higher risk than females.

Regarding the sensitivity analysis restricted to FMs ≥ 50 years (SA4), the highest-risk group included FMs engaged in mixed cattle farming, dairy farming, crop farming, pig farming, cow farming, and truck farming (Supplementary Table 4, Supplementary Figure 4). A positive trend was also observed for viticulture and unspecified and mixed farming. By contrast, the lowest-risk group included FMs engaged in gardening, landscaping and reforestation companies, small animal farming, training, dressage and riding clubs, stud farming, shellfish farming and rural craftsperson. Four activities exhibited a sex difference, with male FMs having a higher risk than females for dairy farming, pig farming, and stud farming; while female FMs engaged in crop farming had a higher risk than males.

Regarding the sensitivity analysis restricted to FMs ≥ 60 years (SA5), the highest-risk group included FMs engaged in dairy farming, mixed cattle farming, pig farming, fruit arboriculture, cow farming, and crop farming (Supplementary Table 4, Supplementary Figure 5). A positive trend was also observed for viticulture and truck farming. By contrast, the lowest-risk group included FMs engaged in stud farming and small animal farming. Three activities exhibited a sex difference, with male FMs having a higher risk than females for fruit arboriculture, pig farming, and stud farming.


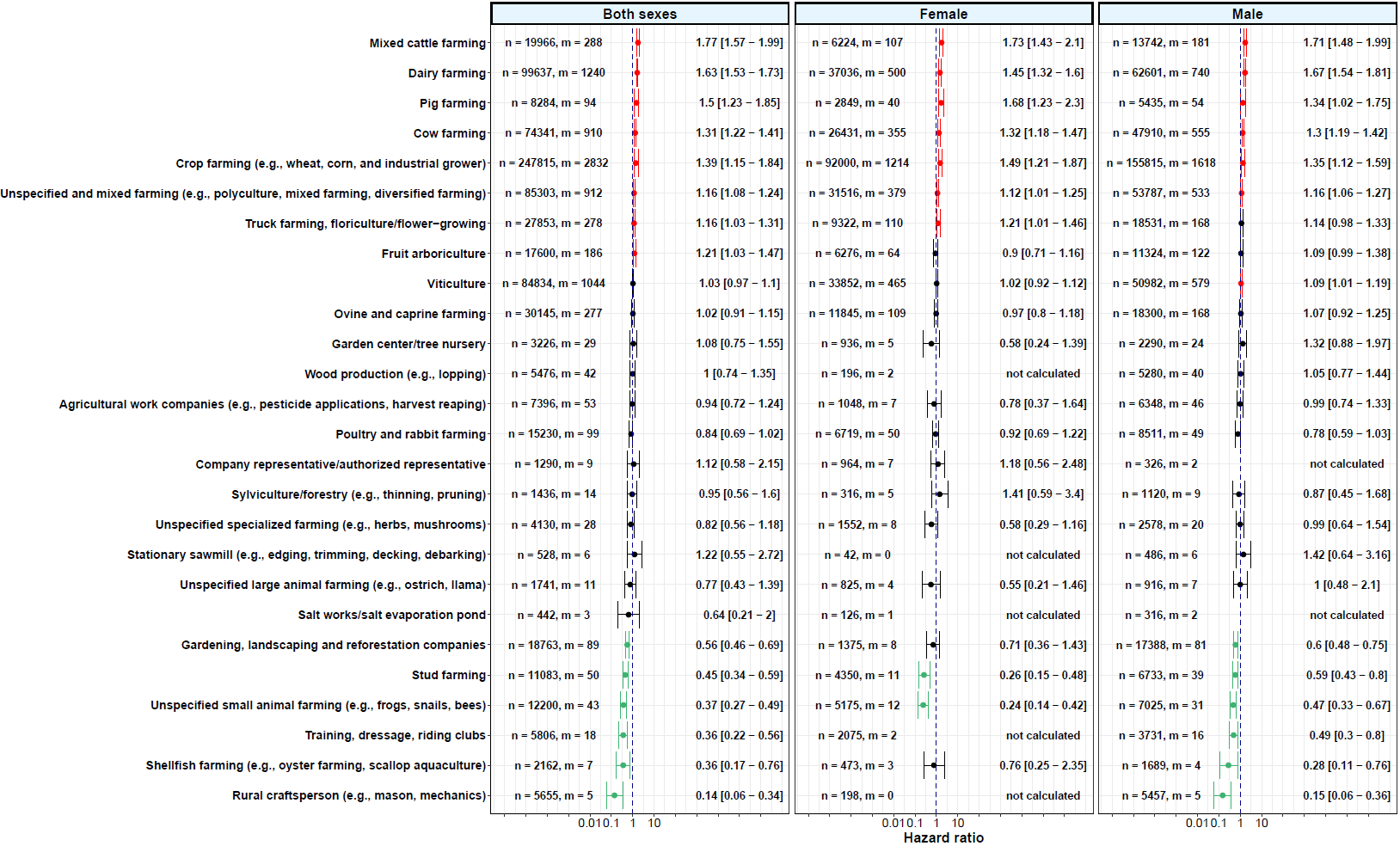


# **Supplementary Figure 3**: Farming activities and risks of Parkinson’s disease, TRACTOR project, 2012-2016 – sensitivity analysis 3

Multivariable Cox regression models for Parkinson’s disease according to each agricultural activity (y-axis) are displayed when the number of exposed cases was sufficient (m ≥ 3). The hazard ratio is represented by a point (x-axis), while error bars represent the 95% confidence interval. The red error bars refer to a higher risk of Parkinson’s disease while the green error bars represent a lower risk of Parkinson’s disease. The black error bars indicate situations where there is no difference in risk of Parkinson’s disease among the farm managers engaged in the considered activity compared to the population of farm managers not performing the considered activity. All analyses were adjusted for sex (for “both sexes” only), age, first year of the farm’s establishment, median farm surface, number of associates, unemployment status, total number of farms, family status, partner work status, farm location, number of comorbidities, and performing a secondary farming activity. n, number of exposed farm managers; m, number of exposed Parkinson’s disease cases.


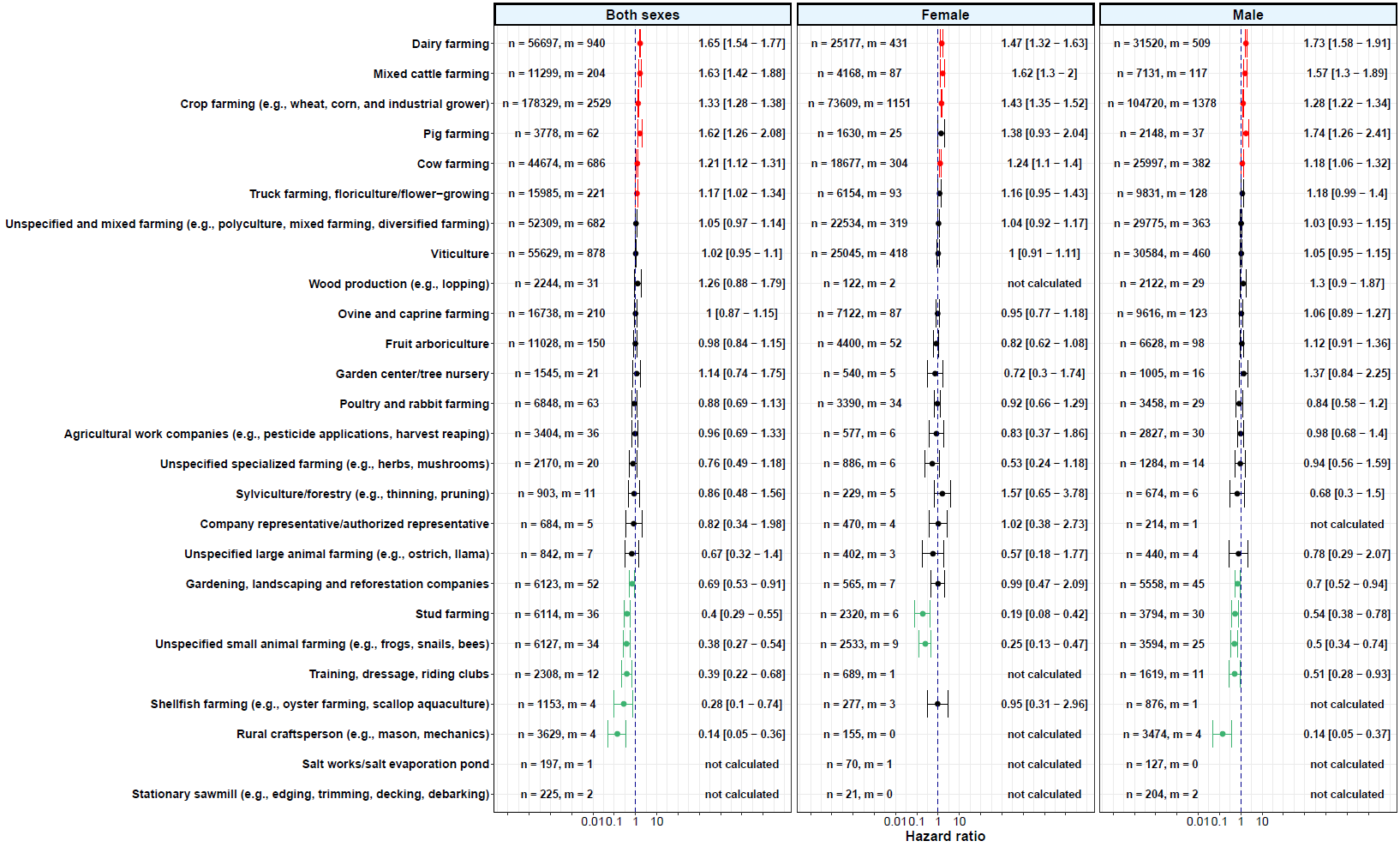


# **Supplementary Figure 4**: Farming activities and risks of Parkinson’s disease, TRACTOR project, 2012-2016 – sensitivity analysis 4

Multivariable Cox regression models for Parkinson’s disease according to each agricultural activity (y-axis) are displayed when the number of exposed cases was sufficient (m ≥ 3). The hazard ratio is represented by a point (x-axis), while error bars represent the 95% confidence interval. The red error bars refer to a higher risk of Parkinson’s disease while the green error bars represent a lower risk of Parkinson’s disease. The black error bars indicate situations where there is no difference in risk of Parkinson’s disease among the farm managers engaged in the considered activity compared to the population of farm managers not performing the considered activity. All analyses were adjusted for sex (for “both sexes” only), age, first year of the farm’s establishment, median farm surface, number of associates, unemployment status, total number of farms, family status, partner work status, farm location, number of comorbidities, and performing a secondary farming activity. n, number of exposed farm managers; m, number of exposed Parkinson’s disease cases.


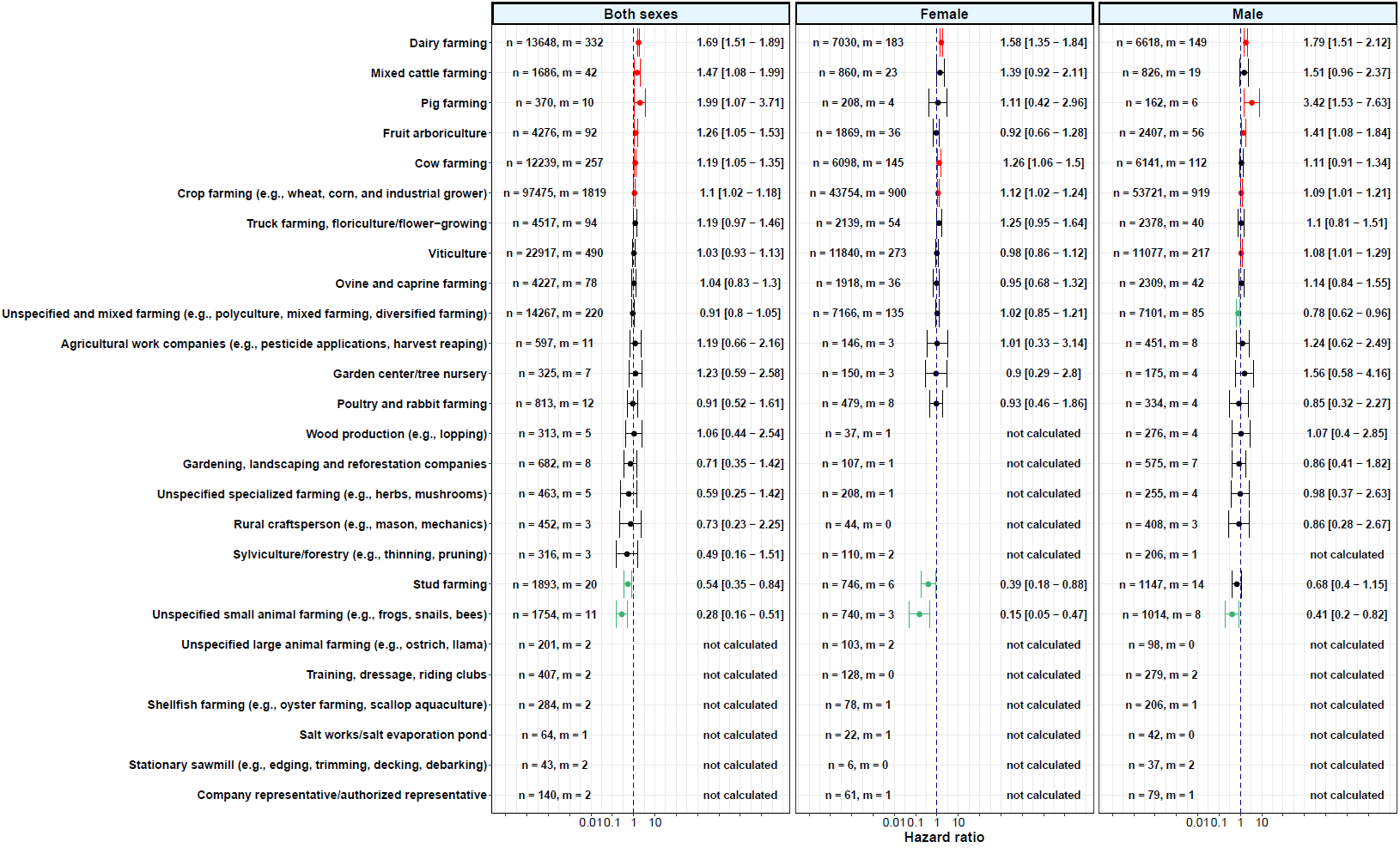


# **Supplementary Figure 5**: Farming activities and risks of Parkinson’s disease, TRACTOR project, 2012-2016 – sensitivity analysis 5

Multivariable Cox regression models for Parkinson’s disease according to each agricultural activity (y-axis) are displayed when the number of exposed cases was sufficient (m ≥ 3). The hazard ratio is represented by a point (x-axis), while error bars represent the 95% confidence interval. The red error bars refer to a higher risk of Parkinson’s disease while the green error bars represent a lower risk of Parkinson’s disease. The black error bars indicate situations where there is no difference in risk of Parkinson’s disease among the farm managers engaged in the considered activity compared to the population of farm managers not performing the considered activity. All analyses were adjusted for sex (for “both sexes” only), age, first year of the farm’s establishment, median farm surface, number of associates, unemployment status, total number of farms, family status, partner work status, farm location, number of comorbidities, and performing a secondary farming activity. n, number of exposed farm managers; m, number of exposed Parkinson’s disease cases.

## *Sensitivity analyses 6 to 14 – addressing* *potential case misclassification*

To address potential case misclassification due to the potential lack of specificity of health data, ten sensitivity analyses with varying stringency were conducted (Supplementary Table 7). For instance, the LTI declaration for G21 (secondary Parkinsonism) was considered as PD cases in several sensitivity analyses. The rationale behind this decision is that, sometimes, differentiating primary Parkinson’s disease (G20) from secondary Parkinsonism (G21) can be challenging, especially in the early stages. If only G20 is used to identify Parkinson’s disease cases, there may be a risk of under-capturing relevant cases that are initially coded as G21 but may later transition to G20. This inclusion could avoid missing cases where secondary parkinsonism was initially misdiagnosed but later identified as primary PD, or where a person exhibits features of both.

The first one, SA2, was described previously.

For SA6, FMs were considered PD cases if they had either at least one LTI (F02, G20, G21) or ODC declaration. The highest-risk group included FMs engaged in crop farming, mixed cattle farming, dairy farming, pig farming, fruit arboriculture, truck farming, unspecified and mixed farming, and cow farming (Supplementary Table 4, Supplementary Figure 6). By contrast, the lowest-risk group included FMs engaged in gardening, landscaping and reforestation companies, small animal farming, and stud farming. Viticulture was the only farming activity exhibiting a sex difference, with male FMs having a higher risk than females.

For SA7, FMs were considered PD cases if they had at least one drug reimbursement of any anti-Parkinson drugs solely used to treat PD. However, FMs only on anticholinergics (trihexyphenidyl, biperiden, and tropatepine) and neuroleptics (drug-induced parkinsonism) were not considered as PD cases. The highest-risk group included FMs engaged in mixed cattle farming, dairy farming, crop farming, cow farming, pig farming, truck farming, unspecified and mixed farming, and viticulture (Supplementary Table 4, Supplementary Figure 7). A positive trend was also observed for ovine and caprine farming. By contrast, the lowest-risk group included FMs engaged in gardening, landscaping and reforestation companies, small animal farming, training, dressage and riding clubs, stud farming, shellfish farming and rural craftsperson. Pig farming was the only farming activity exhibiting a sex difference, with female FMs having a higher risk than males.

For SA8, FMs who had at least one drug reimbursement of any anti-Parkinson drugs solely used to treat PD and also a LTI declaration for Alzheimer’s disease and other related dementias (ADRD) that corresponded to ICD-10 codes F00 (“dementia in Alzheimer’s disease”), F01 (“vascular dementia”), F02 (“dementia in other diseases classified elsewhere”), F03 (“unspecified dementia”), and G30 (“Alzheimer’s disease”) were not considered PD cases. In addition, FMs only on anticholinergics (trihexyphenidyl, biperiden, and tropatepine) and neuroleptics (drug-induced parkinsonism) were not considered as PD cases. The highest-risk group included FMs engaged in mixed cattle farming, crop farming, dairy farming, pig farming, cow farming, unspecified and mixed farming, and truck farming (Supplementary Table 4, Supplementary Figure 8). A positive trend was also observed for viticulture and ovine and caprine farming. By contrast, the lowest-risk group included FMs engaged in gardening, landscaping and reforestation companies, small animal farming, training, dressage and riding clubs, stud farming, shellfish farming and rural craftsperson. No activity exhibited a sex difference.

For SA9, FMs were considered PD cases if they had one LTI declaration with either the ICD-10 code G20 (“Parkinson’s disease”) or G21 (“secondary parkinsonism”). The highest-risk group included FMs engaged in crop farming, mixed cattle farming, dairy farming, pig farming, fruit arboriculture, cow farming, unspecified and mixed farming, and truck farming (Supplementary Table 4, Supplementary Figure 9). A positive trend was also observed for viticulture and sylviculture. By contrast, the lowest-risk group included FMs engaged in gardening, landscaping and reforestation companies, small animal farming, training, dressage and riding clubs, and stud farming. Only viticulture exhibited a sex difference, with male FMs having a higher risk than females.

For SA10, FMs were considered PD cases if they had one LTI declaration with either the ICD-10 code F02, G20, or G21. The highest-risk group included FMs engaged in crop farming, mixed cattle farming, dairy farming, fruit arboriculture, viticulture, pig farming, cow farming, unspecified and mixed farming, and truck farming (Supplementary Table 4, Supplementary Figure 10). A positive trend was also observed for sylviculture. By contrast, the lowest-risk group included FMs engaged in gardening, landscaping and reforestation companies, small animal farming, and stud farming. Only viticulture exhibited a sex difference, with male FMs having a higher risk than females.

For SA11, FMs were considered PD cases if they had either at least one LTI declaration (F02, G20, G21), one ODC declaration, or one drug reimbursement of any anti-Parkinson drugs even, if the drug is not solely used for PD. The highest-risk group included FMs engaged in crop farming, dairy farming, mixed cattle farming, pig farming, fruit arboriculture, cow farming, viticulture, unspecified and mixed farming, and truck farming (Supplementary Table 4, Supplementary Figure 11). A positive trend was also observed for ovine and caprine farming and wood production. By contrast, the lowest-risk group included FMs engaged in small animal farming, training, dressage and riding clubs, stud farming, and rural craftsperson. Three activities exhibited a sex difference, with male FMs having a higher risk than females for viticulture and stud farming; while female FMs engaged in mixed cattle farming had a higher risk than males.

For SA12, FMs were considered PD cases if they had either at least one LTI (F02, G20), one ODC declaration, or two drug reimbursements of any anti-Parkinson drugs solely used to treat PD. However, FMs only on anticholinergics (trihexyphenidyl, biperiden, and tropatepine) and neuroleptics (drug-induced parkinsonism) were not considered as PD cases. The highest-risk group included FMs engaged in crop farming, dairy farming, mixed cattle farming, cow farming, pig farming, truck farming, and fruit arboriculture (Supplementary Table 4, Supplementary Figure 12). A positive trend was also observed for unspecified and mixed farming, viticulture, and ovine and caprine farming. By contrast, the lowest-risk group included FMs engaged in gardening, landscaping and reforestation companies, small animal farming, training, dressage and riding clubs, stud farming, and rural craftsperson. Viticulture and stud farming were the only two farming activities exhibiting a sex difference, with male FMs having a higher risk than females.

For SA13, FMs were considered PD cases if they had either at least one LTI (F02, G20), one ODC declaration, or three drug reimbursements of any anti-Parkinson drugs solely used to treat PD. However, FMs only on anticholinergics (trihexyphenidyl, biperiden, and tropatepine) and neuroleptics (drug-induced parkinsonism) were not considered as PD cases. The highest-risk group included FMs engaged in crop farming, dairy farming, mixed cattle farming, pig farming, cow farming, truck farming, and fruit arboriculture (Supplementary Table 4, Supplementary Figure 13). A positive trend was also observed for unspecified and mixed farming, viticulture, and ovine and caprine farming. By contrast, the lowest-risk group included FMs engaged in gardening, landscaping and reforestation companies, small animal farming, training, dressage and riding clubs, and stud farming. A negative trend was also observed for shellfish farming. Viticulture, stud farming and gardening, landscaping and reforestation companies were the only farming activities exhibiting a sex difference, with male FMs having a higher risk than females, with the exception of gardening, landscaping and reforestation companies.

For SA14, FMs were considered PD cases if they had either at least one LTI (F02, G20), one ODC declaration, or six drug reimbursements of any anti-Parkinson drugs solely used to treat PD. However, FMs only on anticholinergics (trihexyphenidyl, biperiden, and tropatepine) and neuroleptics (drug-induced parkinsonism) were not considered as PD cases. The highest-risk group included FMs engaged in dairy farming, crop farming, mixed cattle farming, pig farming, cow farming, fruit arboriculture, and unspecified and mixed farming (Supplementary Table 4, Supplementary Figure 14). A positive trend was also observed for truck farming, and viticulture. By contrast, the lowest-risk group included FMs engaged in poultry and rabbit farming, gardening, landscaping and reforestation companies, small animal farming, training, dressage and riding clubs, and stud farming. Viticulture and training, dressage, riding clubs were the only farming activities exhibiting a sex difference, with male FMs having a higher risk than females. A trend was also observed for unspecified specialized farming and stud farming, with risk higher for males than females. In contrast, a trend with higher risk among females than males was observed for both truck farming and shellfish farming.


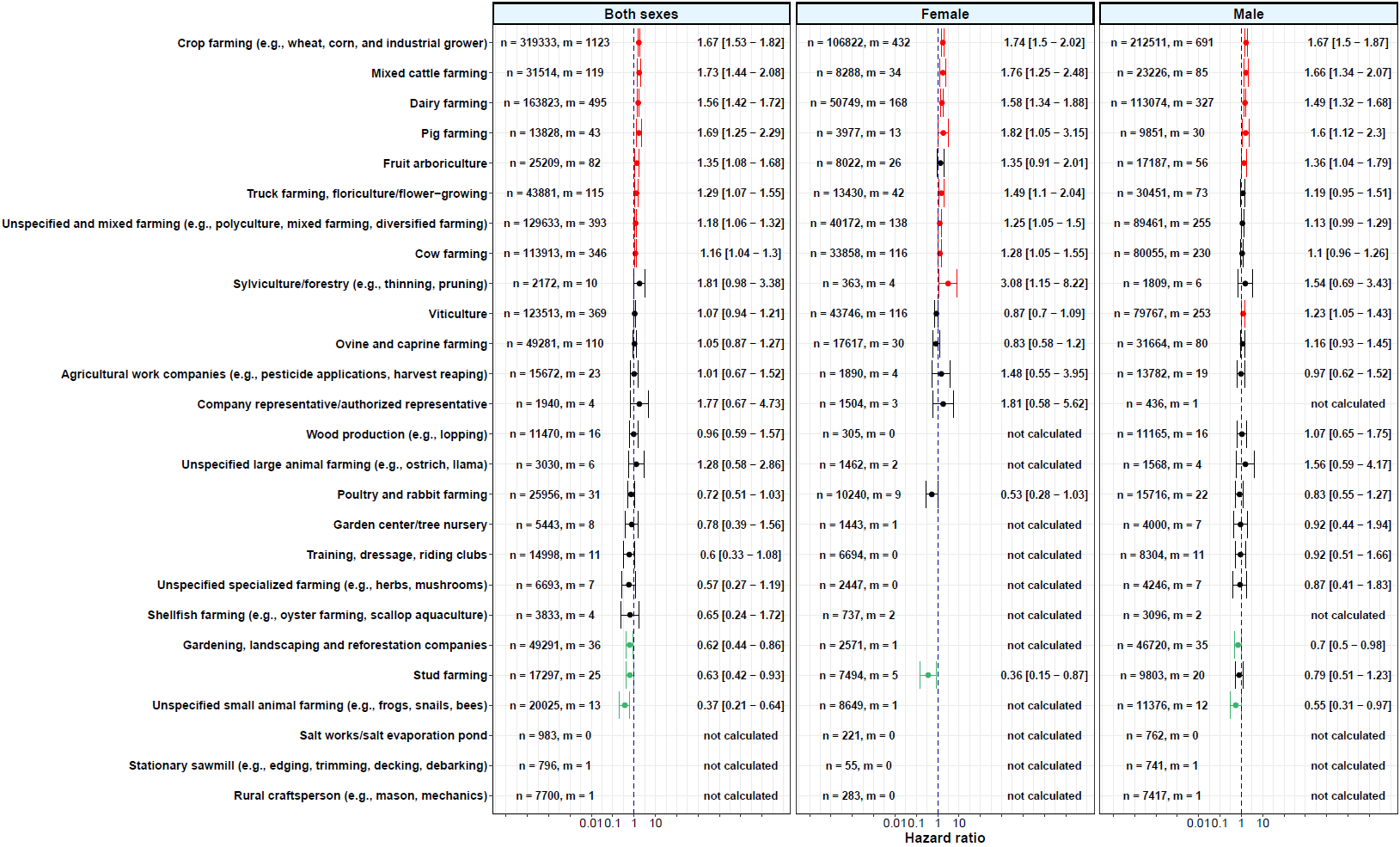


# **Supplementary Figure 6**: Farming activities and risks of Parkinson’s disease, TRACTOR project, 2012-2016 – sensitivity analysis 6

Multivariable Cox regression models for Parkinson’s disease according to each agricultural activity (y-axis) are displayed when the number of exposed cases was sufficient (m ≥ 3). The hazard ratio is represented by a point (x-axis), while error bars represent the 95% confidence interval. The red error bars refer to a higher risk of Parkinson’s disease while the green error bars represent a lower risk of Parkinson’s disease. The black error bars indicate situations where there is no difference in risk of Parkinson’s disease among the farm managers engaged in the considered activity compared to the population of farm managers not performing the considered activity. All analyses were adjusted for sex (for “both sexes” only), age, first year of the farm’s establishment, median farm surface, number of associates, unemployment status, total number of farms, family status, partner work status, farm location, number of comorbidities, and performing a secondary farming activity. n, number of exposed farm managers; m, number of exposed Parkinson’s disease cases.


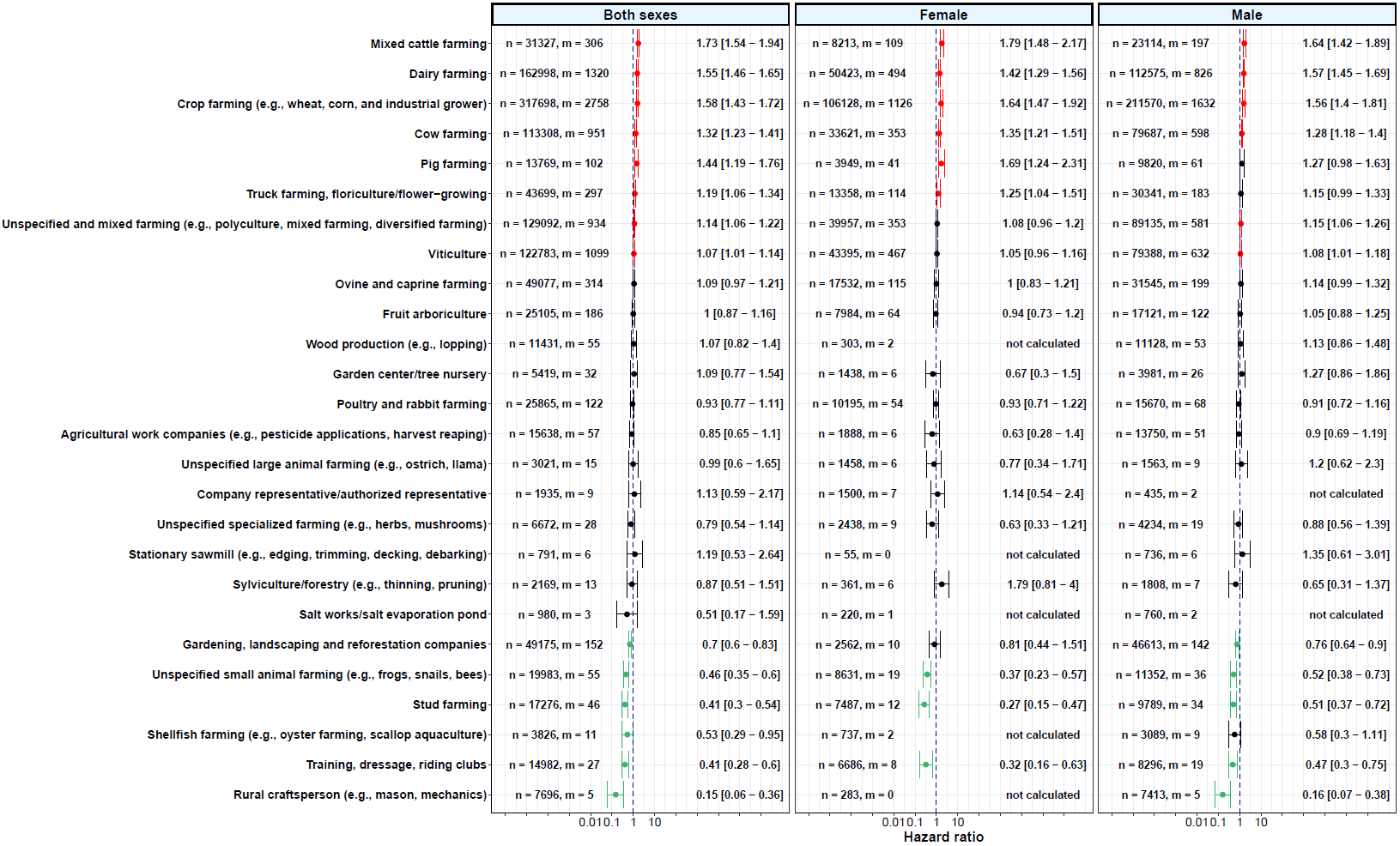


# **Supplementary Figure 7**: Farming activities and risks of Parkinson’s disease, TRACTOR project, 2012-2016 – sensitivity analysis 7

Multivariable Cox regression models for Parkinson’s disease according to each agricultural activity (y-axis) are displayed when the number of exposed cases was sufficient (m ≥ 3). The hazard ratio is represented by a point (x-axis), while error bars represent the 95% confidence interval. The red error bars refer to a higher risk of Parkinson’s disease while the green error bars represent a lower risk of Parkinson’s disease. The black error bars indicate situations where there is no difference in risk of Parkinson’s disease among the farm managers engaged in the considered activity compared to the population of farm managers not performing the considered activity. All analyses were adjusted for sex (for “both sexes” only), age, first year of the farm’s establishment, median farm surface, number of associates, unemployment status, total number of farms, family status, partner work status, farm location, number of comorbidities, and performing a secondary farming activity. n, number of exposed farm managers; m, number of exposed Parkinson’s disease cases.


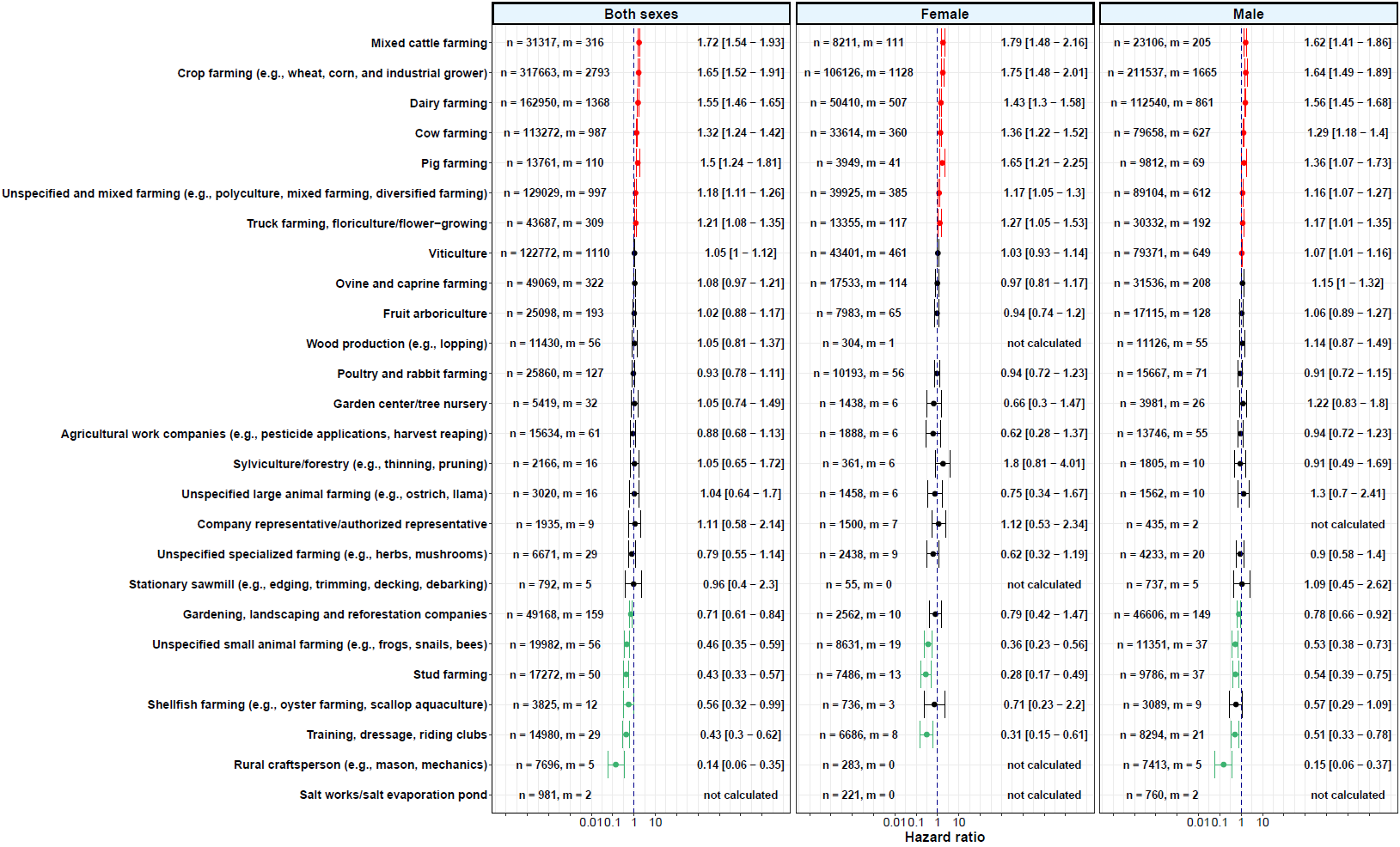


# **Supplementary Figure 8**: Farming activities and risks of Parkinson’s disease, TRACTOR project, 2012-2016 – sensitivity analysis 8

Multivariable Cox regression models for Parkinson’s disease according to each agricultural activity (y-axis) are displayed when the number of exposed cases was sufficient (m ≥ 3). The hazard ratio is represented by a point (x-axis), while error bars represent the 95% confidence interval. The red error bars refer to a higher risk of Parkinson’s disease while the green error bars represent a lower risk of Parkinson’s disease. The black error bars indicate situations where there is no difference in risk of Parkinson’s disease among the farm managers engaged in the considered activity compared to the population of farm managers not performing the considered activity. All analyses were adjusted for sex (for “both sexes” only), age, first year of the farm’s establishment, median farm surface, number of associates, unemployment status, total number of farms, family status, partner work status, farm location, number of comorbidities, and performing a secondary farming activity. n, number of exposed farm managers; m, number of exposed Parkinson’s disease cases.


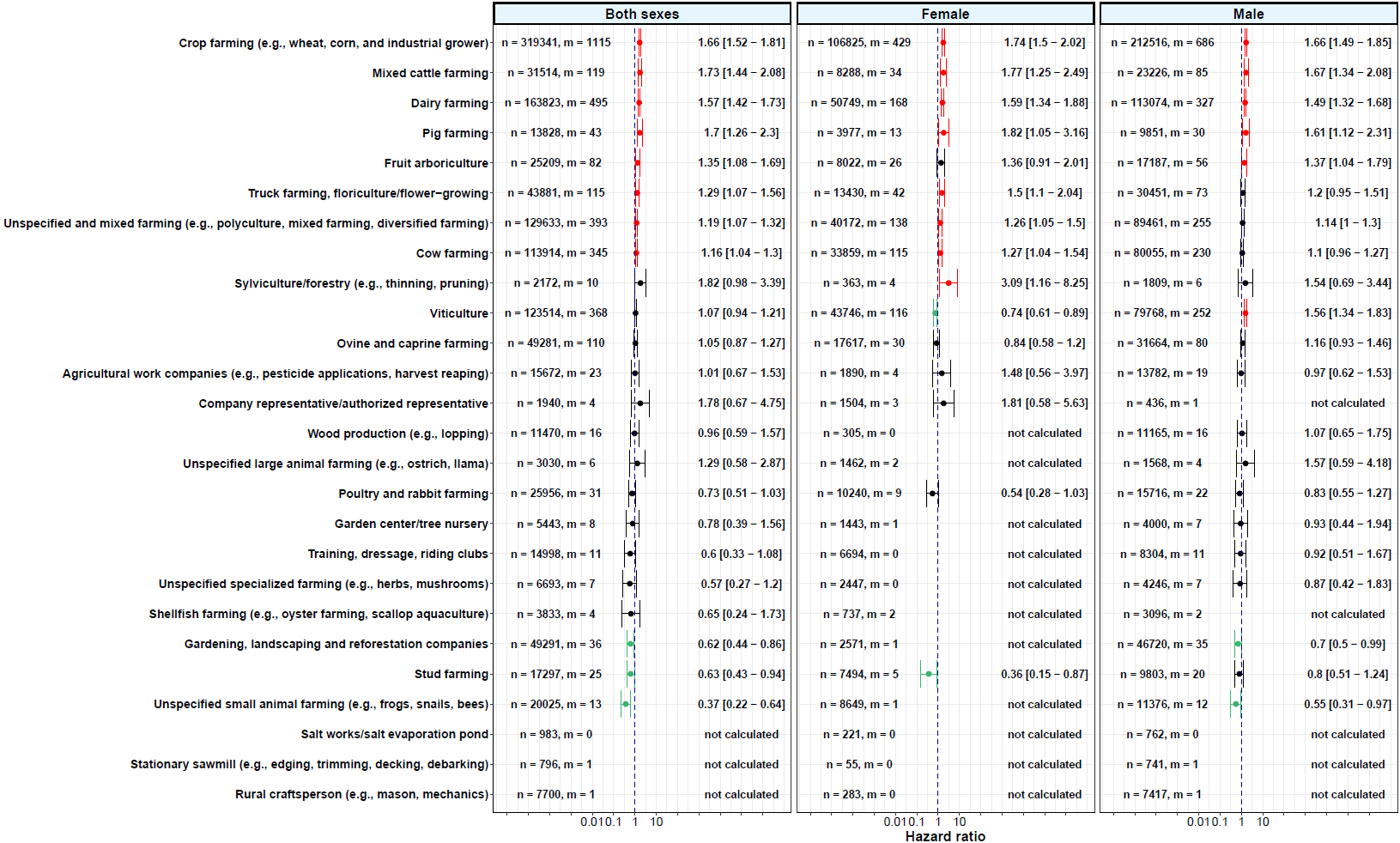


# **Supplementary Figure 9**: Farming activities and risks of Parkinson’s disease, TRACTOR project, 2012-2016 – sensitivity analysis 9

Multivariable Cox regression models for Parkinson’s disease according to each agricultural activity (y-axis) are displayed when the number of exposed cases was sufficient (m ≥ 3). The hazard ratio is represented by a point (x-axis), while error bars represent the 95% confidence interval. The red error bars refer to a higher risk of Parkinson’s disease while the green error bars represent a lower risk of Parkinson’s disease. The black error bars indicate situations where there is no difference in risk of Parkinson’s disease among the farm managers engaged in the considered activity compared to the population of farm managers not performing the considered activity. All analyses were adjusted for sex (for “both sexes” only), age, first year of the farm’s establishment, median farm surface, number of associates, unemployment status, total number of farms, family status, partner work status, farm location, number of comorbidities, and performing a secondary farming activity. n, number of exposed farm managers; m, number of exposed Parkinson’s disease cases.


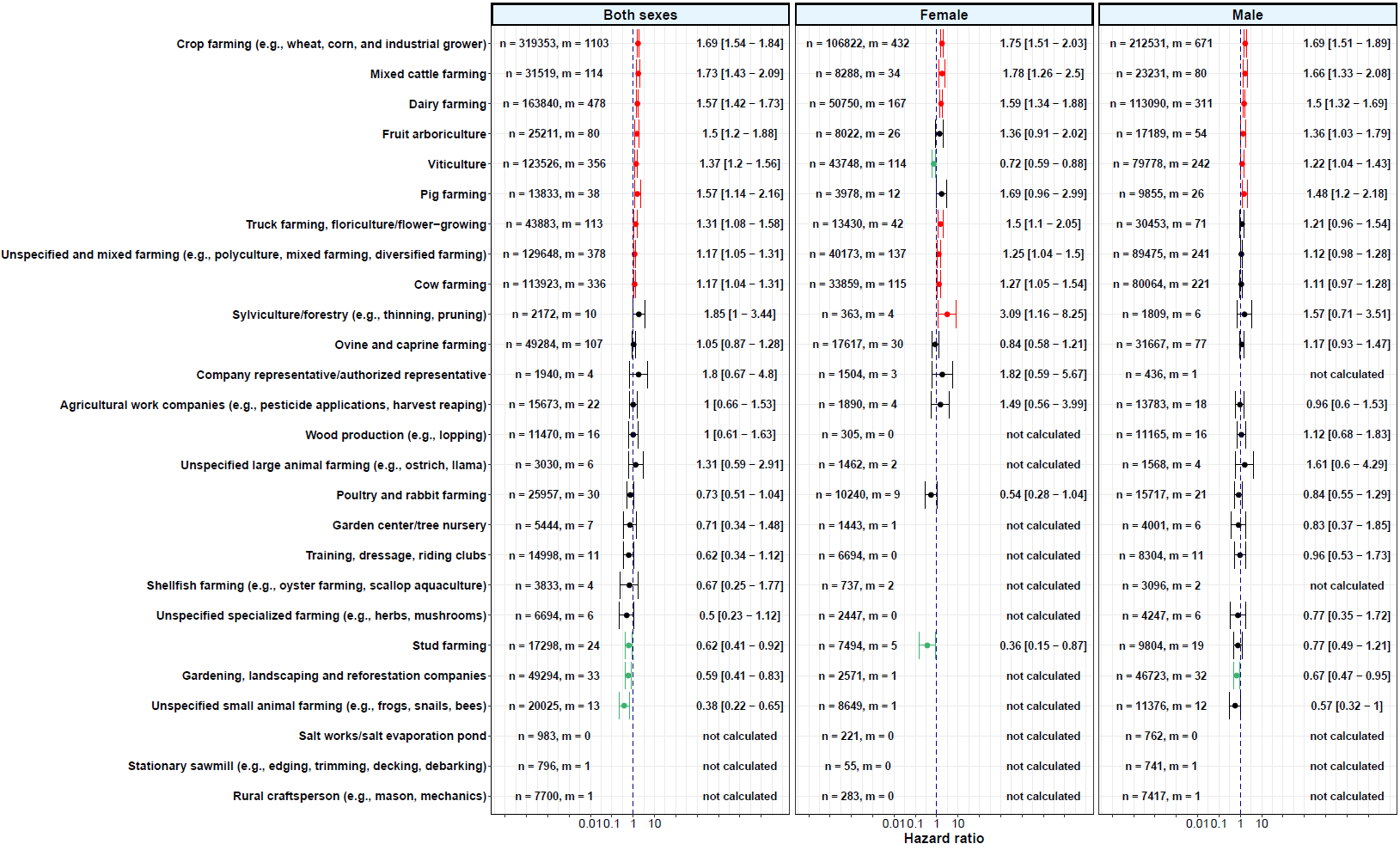


# **Supplementary Figure 10**: Farming activities and risks of Parkinson’s disease, TRACTOR project, 2012-2016 – sensitivity analysis 10

Multivariable Cox regression models for Parkinson’s disease according to each agricultural activity (y-axis) are displayed when the number of exposed cases was sufficient (m ≥ 3). The hazard ratio is represented by a point (x-axis) while error bars represent the 95% confidence interval. The red error bars refer to a higher risk of Parkinson’s disease, while the green error bars represent a lower risk of Parkinson’s disease. The black error bars indicate situations where there is no difference in risk of Parkinson’s disease among the farm managers engaged in the considered activity compared to the population of farm managers not performing the considered activity. All analyses were adjusted for sex (for “both sexes” only), age, first year of the farm’s establishment, median farm surface, number of associates, unemployment status, total number of farms, family status, partner work status, farm location, number of comorbidities, and performing a secondary farming activity. n, number of exposed farm managers; m, number of exposed Parkinson’s disease cases.


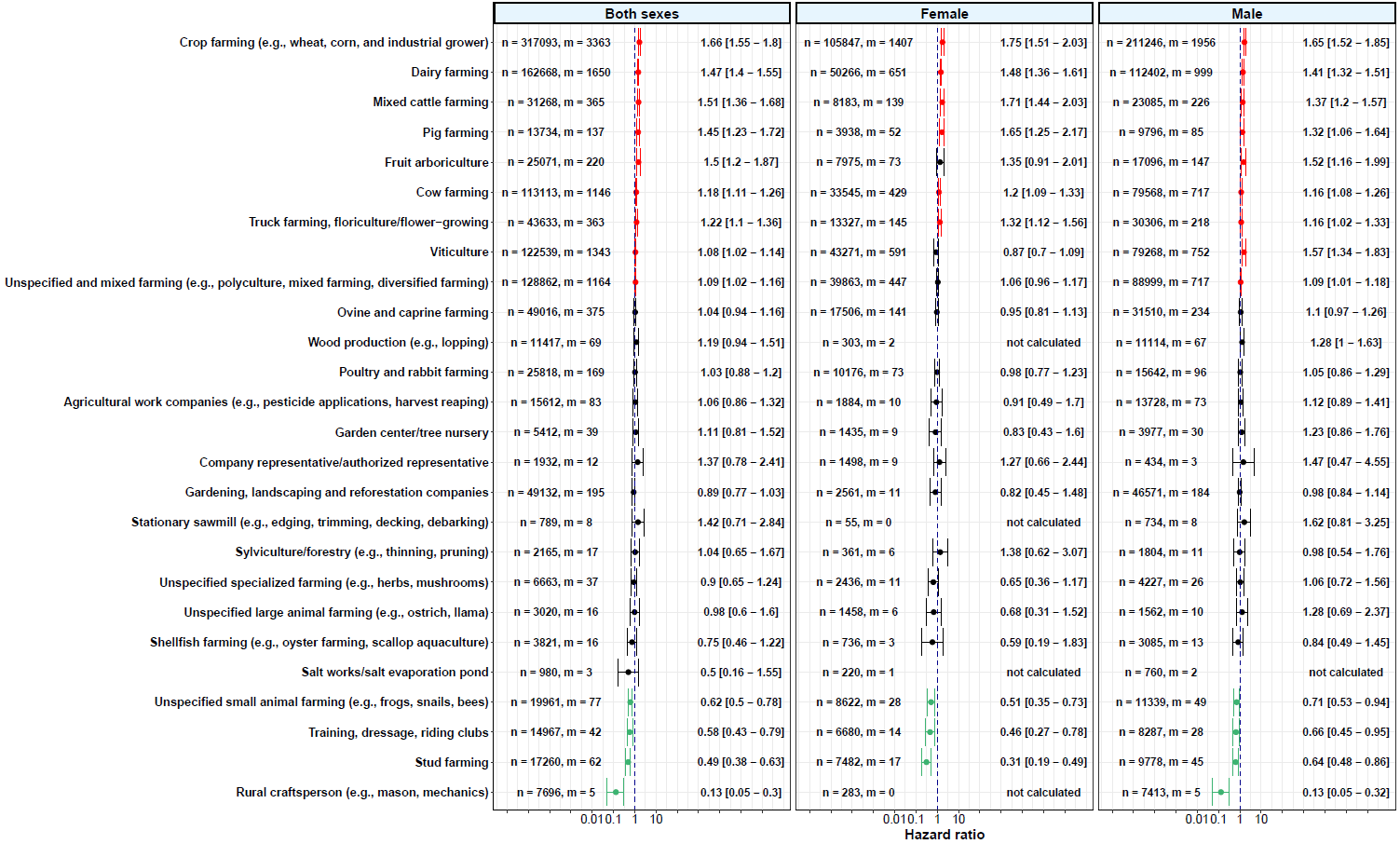


# **Supplementary Figure 11**: Farming activities and risks of Parkinson’s disease, TRACTOR project, 2012-2016 – sensitivity analysis 11

Multivariable Cox regression models for Parkinson’s disease according to each agricultural activity (y-axis) are displayed when the number of exposed cases was sufficient (m ≥ 3). The hazard ratio is represented by a point (x-axis), while error bars represent the 95% confidence interval. The red error bars refer to a higher risk of Parkinson’s disease while the green error bars represent a lower risk of Parkinson’s disease. The black error bars indicate situations where there is no difference in risk of Parkinson’s disease among the farm managers engaged in the considered activity compared to the population of farm managers not performing the considered activity. All analyses were adjusted for sex (for “both sexes” only), age, first year of the farm’s establishment, median farm surface, number of associates, unemployment status, total number of farms, family status, partner work status, farm location, number of comorbidities, and performing a secondary farming activity. n, number of exposed farm managers; m, number of exposed Parkinson’s disease cases.


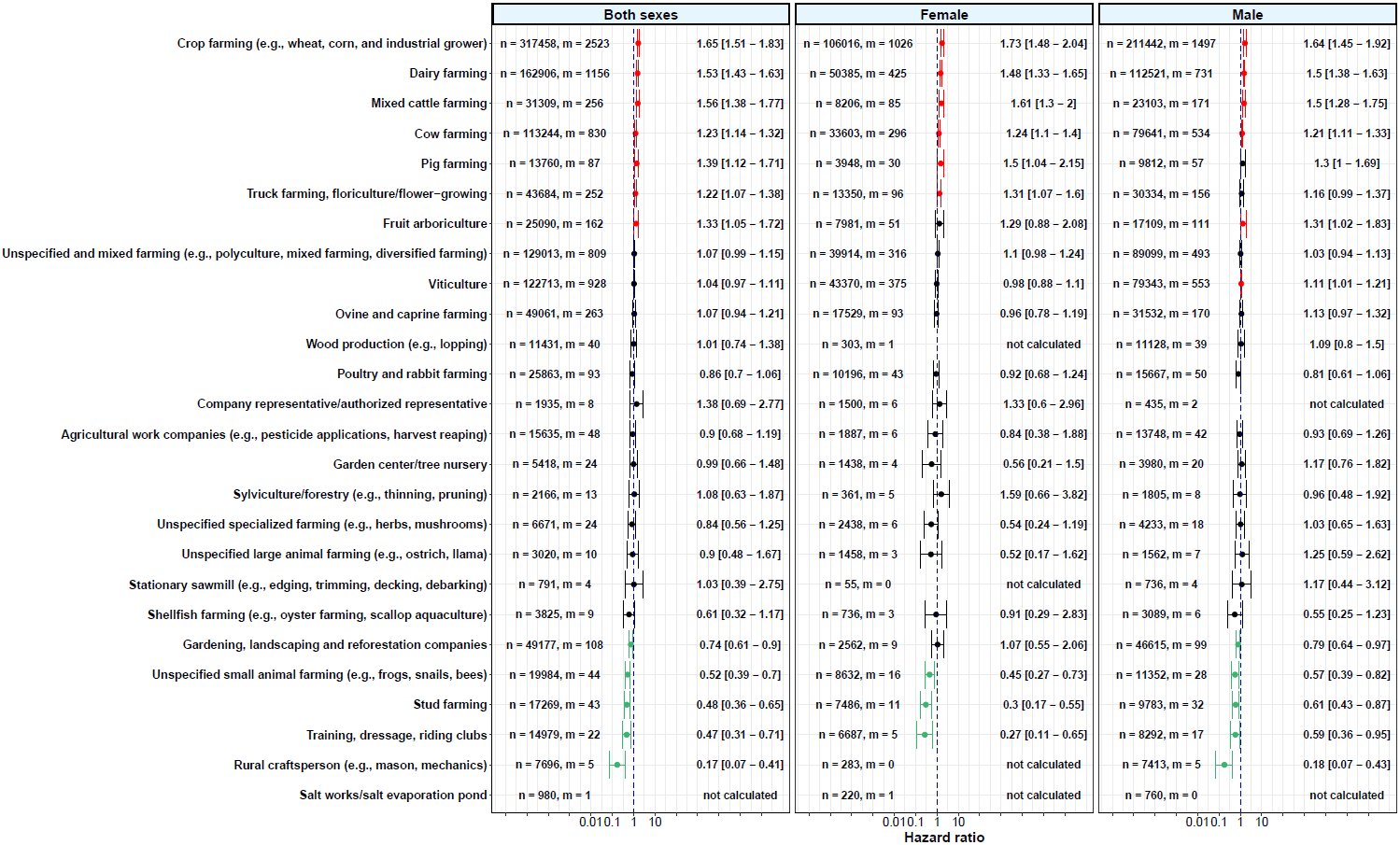


# **Supplementary Figure 12**: Farming activities and risks of Parkinson’s disease, TRACTOR project, 2012-2016 – sensitivity analysis 12

Multivariable Cox regression models for Parkinson’s disease according to each agricultural activity (y-axis) are displayed when the number of exposed cases was sufficient (m ≥ 3). The hazard ratio is represented by a point (x-axis), while error bars represent the 95% confidence interval. The red error bars refer to a higher risk of Parkinson’s disease while the green error bars represent a lower risk of Parkinson’s disease. The black error bars indicate situations where there is no difference in risk of Parkinson’s disease among the farm managers engaged in the considered activity compared to the population of farm managers not performing the considered activity. All analyses were adjusted for sex (for “both sexes” only), age, first year of the farm’s establishment, median farm surface, number of associates, unemployment status, total number of farms, family status, partner work status, farm location, number of comorbidities, and performing a secondary farming activity. n, number of exposed farm managers; m, number of exposed Parkinson’s disease cases.


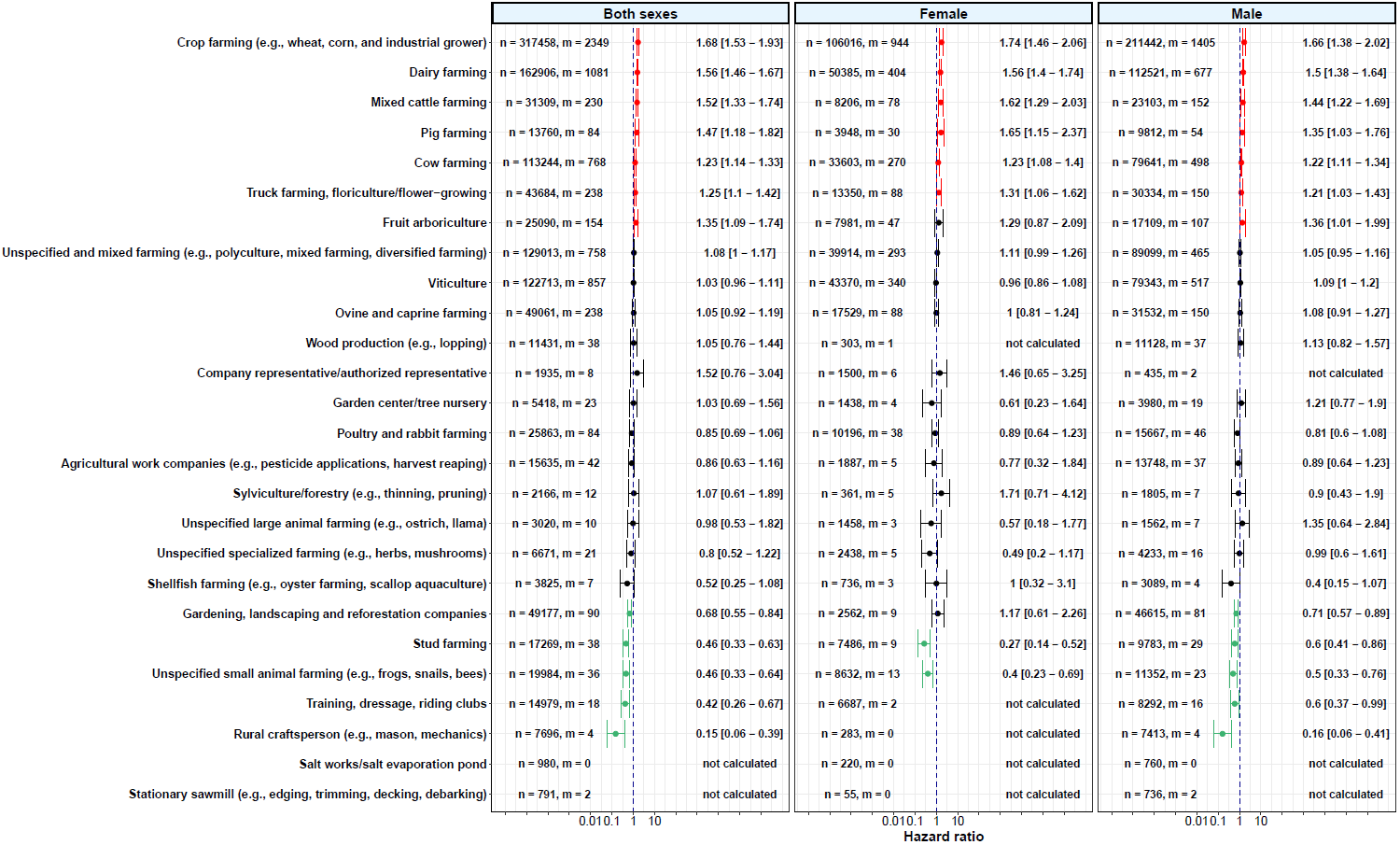


# **Supplementary Figure 13**: Farming activities and risks of Parkinson’s disease, TRACTOR project, 2012-2016 – sensitivity analysis 13

Multivariable Cox regression models for Parkinson’s disease according to each agricultural activity (y-axis) are displayed when the number of exposed cases was sufficient (m ≥ 3). The hazard ratio is represented by a point (x-axis), while error bars represent the 95% confidence interval. The red error bars refer to a higher risk of Parkinson’s disease while the green error bars represent a lower risk of Parkinson’s disease. The black error bars indicate situations where there is no difference in risk of Parkinson’s disease among the farm managers engaged in the considered activity compared to the population of farm managers not performing the considered activity. All analyses were adjusted for sex (for “both sexes” only), age, first year of the farm’s establishment, median farm surface, number of associates, unemployment status, total number of farms, family status, partner work status, farm location, number of comorbidities, and performing a secondary farming activity. n, number of exposed farm managers; m, number of exposed Parkinson’s disease cases.


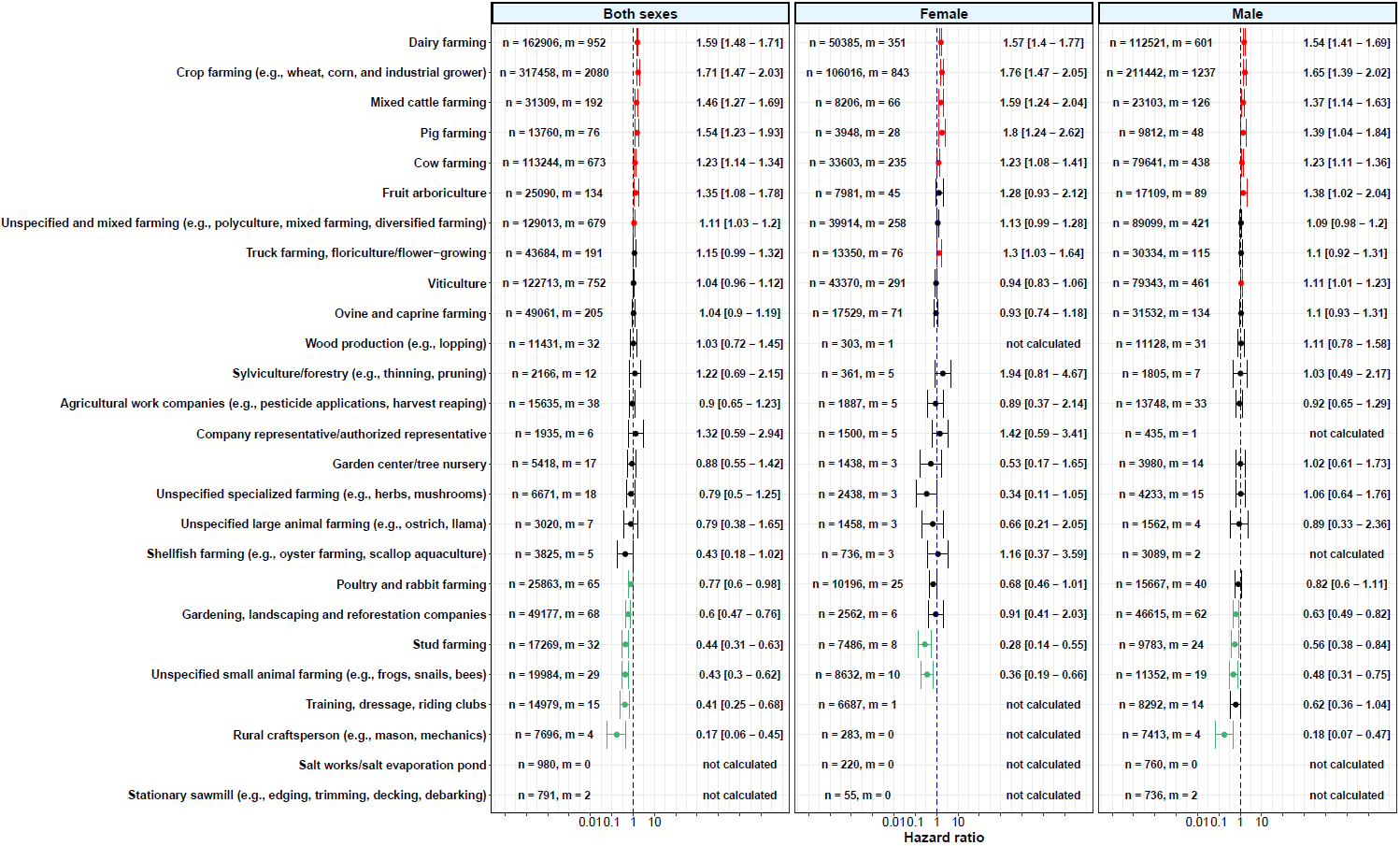


# **Supplementary Figure 14**: Farming activities and risks of Parkinson’s disease, TRACTOR project, 2012-2016 – sensitivity analysis 14

Multivariable Cox regression models for Parkinson’s disease according to each agricultural activity (y-axis) are displayed when the number of exposed cases was sufficient (m ≥ 3). The hazard ratio is represented by a point (x-axis), while error bars represent the 95% confidence interval. The red error bars refer to a higher risk of Parkinson’s disease while the green error bars represent a lower risk of Parkinson’s disease. The black error bars indicate situations where there is no difference in risk of Parkinson’s disease among the farm managers engaged in the considered activity compared to the population of farm managers not performing the considered activity. All analyses were adjusted for sex (for “both sexes” only), age, first year of the farm’s establishment, median farm surface, number of associates, unemployment status, total number of farms, family status, partner work status, farm location, number of comorbidities, and performing a secondary farming activity. n, number of exposed farm managers; m, number of exposed Parkinson’s disease cases.

## *Sensitivity analysis 15 – using only one farming activity as reference*

In the main analysis and other sensitivity analyses, we considered farm managers to be exposed to a given farming activity if they had been engaged in this activity at least once between 2002 and 2016 (1 yearly declaration to MSA). Instead, in this sensitivity analysis, each farm manager was considered to be engaged solely in their longest farming activity ever practiced in terms of number of years. In case the longest exposure corresponded to several farming activities (e.g., 4 years as a dairy farmer, then 4 years as crop farmer), only the oldest farming activity (e.g., dairy farming in the previous example) was considered.

Similarly to the main analysis, for this sensitivity analysis, FMs were considered to have PD if they had at least one LTI declaration for PD (ICD-10 code G20 or F02), one ODC declaration for PD, or one reimbursement of any drugs solely used to treat PD (i.e., all antiparkinsonian agents, with the exception of pramipexole, rotigotine, amantadine, and lisuride) (Supplementary Table 7). However, FMs only on anticholinergics (trihexyphenidyl, biperiden, and tropatepine) and neuroleptics (drug-induced parkinsonism) were not considered PD cases.

The highest-risk group included FMs engaged in mixed cattle farming, dairy farming, crop farming, fruit arboriculture, cow farming, sylviculture, truck farming, pig farming, and viticulture (Supplementary Table 4, Supplementary Figure 15). By contrast, the lowest-risk group included FMs engaged in gardening, landscaping and reforestation companies, small animal farming, training, dressage and riding clubs, stud farming, and rural craftsperson. Two activities exhibited a sex difference, with male FMs having a higher risk than females for viticulture and stud farming.


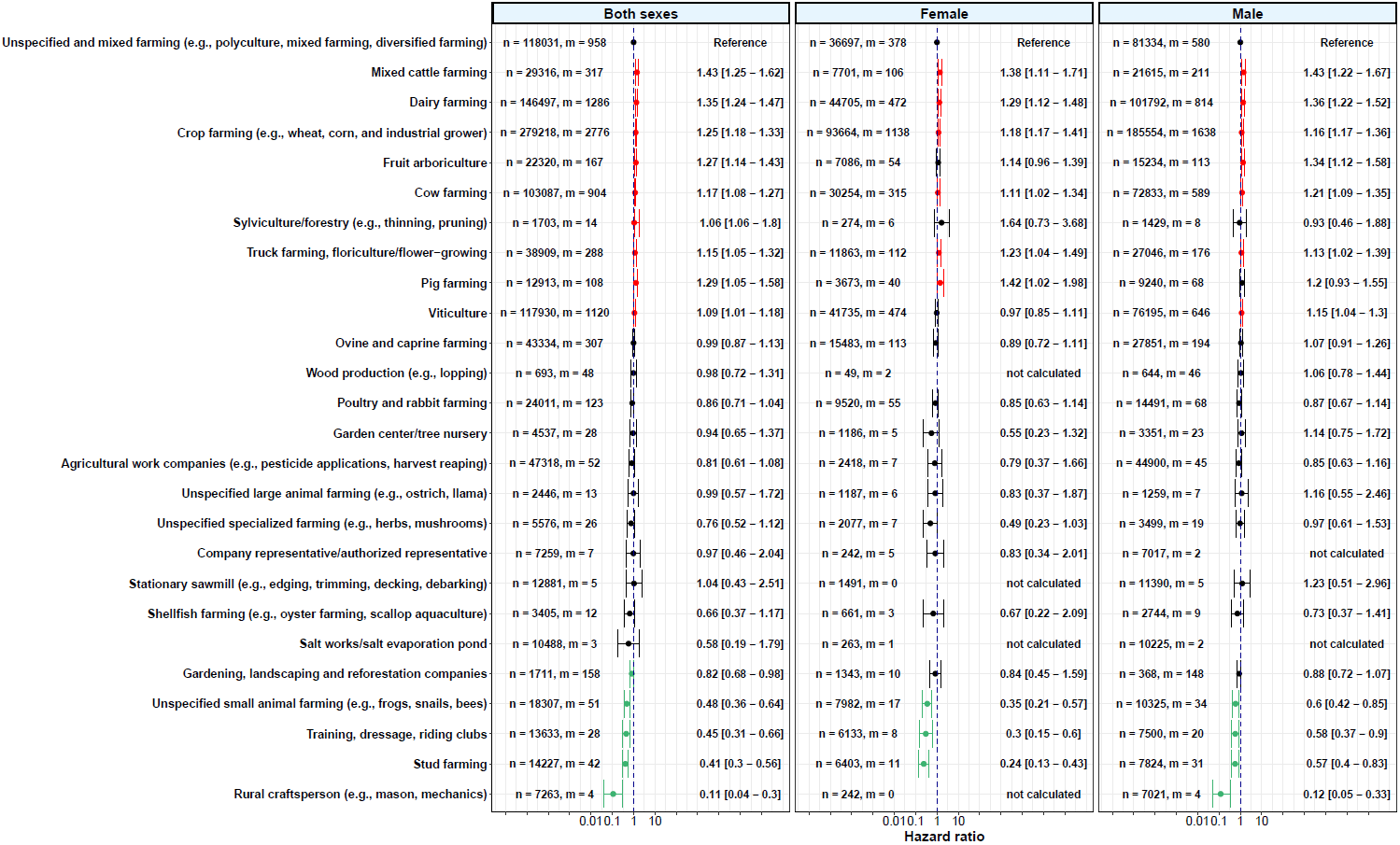


# **Supplementary Figure 15**: Farming activities and risks of Parkinson’s disease, TRACTOR project, 2012-2016 – sensitivity analysis 15

Multivariable Cox regression models for Parkinson’s disease according to each agricultural activity (y-axis) are displayed when the number of exposed cases was sufficient (m ≥ 3). The hazard ratio is represented by a point (x-axis), while error bars represent the 95% confidence interval. The red error bars refer to a higher risk of Parkinson’s disease while the green error bars represent a lower risk of Parkinson’s disease. The black error bars indicate situations where there is no difference in risk of Parkinson’s disease among the farm managers engaged in the considered activity compared to the population of farm managers not performing the considered activity. All analyses were adjusted for sex (for “both sexes” only), age, first year of the farm’s establishment, median farm surface, number of associates, unemployment status, total number of farms, family status, partner work status, farm location, number of comorbidities, and performing a secondary farming activity. n, number of exposed farm managers; m, number of exposed Parkinson’s disease cases.

## *Sensitivity analysis 16 – adjusting for smoking*

Because we did not have smoking data, we performed a sensitivity analysis adjusting for smoking. To that end, we randomly generated the smoking status (current smokers/non-smokers) by assuming that there was a prevalence of current smokers of 19% for crop farming, 9% for cattle farming, 6% for pig farming, 15% for poultry farming, and 18% for all other farming activities. These prevalences were based on a cross-sectional study conducted in 3787 French farmers affiliated with MSA in 2013^4^.

Similarly to the main analysis, for this sensitivity analysis, FMs were considered to have PD if they had at least one LTI declaration for PD (ICD-10 code G20 or F02), one ODC declaration for PD, or one reimbursement of any drugs solely used to treat PD (i.e., all antiparkinsonian agents, with the exception of pramipexole, rotigotine, amantadine, and lisuride) (Supplementary Table 7). However, FMs only on anticholinergics (trihexyphenidyl, biperiden, and tropatepine) and neuroleptics (drug-induced parkinsonism) were not considered PD cases.

The highest-risk group included FMs engaged in crop farming, mixed cattle farming, dairy farming, pig farming, cow farming, truck farming, fruit arboriculture, unspecified and mixed farming, and viticulture (Supplementary Table 4, Supplementary Figure 16). A positive trend was also observed for ovine and caprine farming. By contrast, the lowest-risk group included FMs engaged in small animal farming, training, dressage and riding clubs, stud farming, and rural craftsperson. Stud farming was the only farming activity exhibiting a sex difference, with male FMs having a higher risk than females.


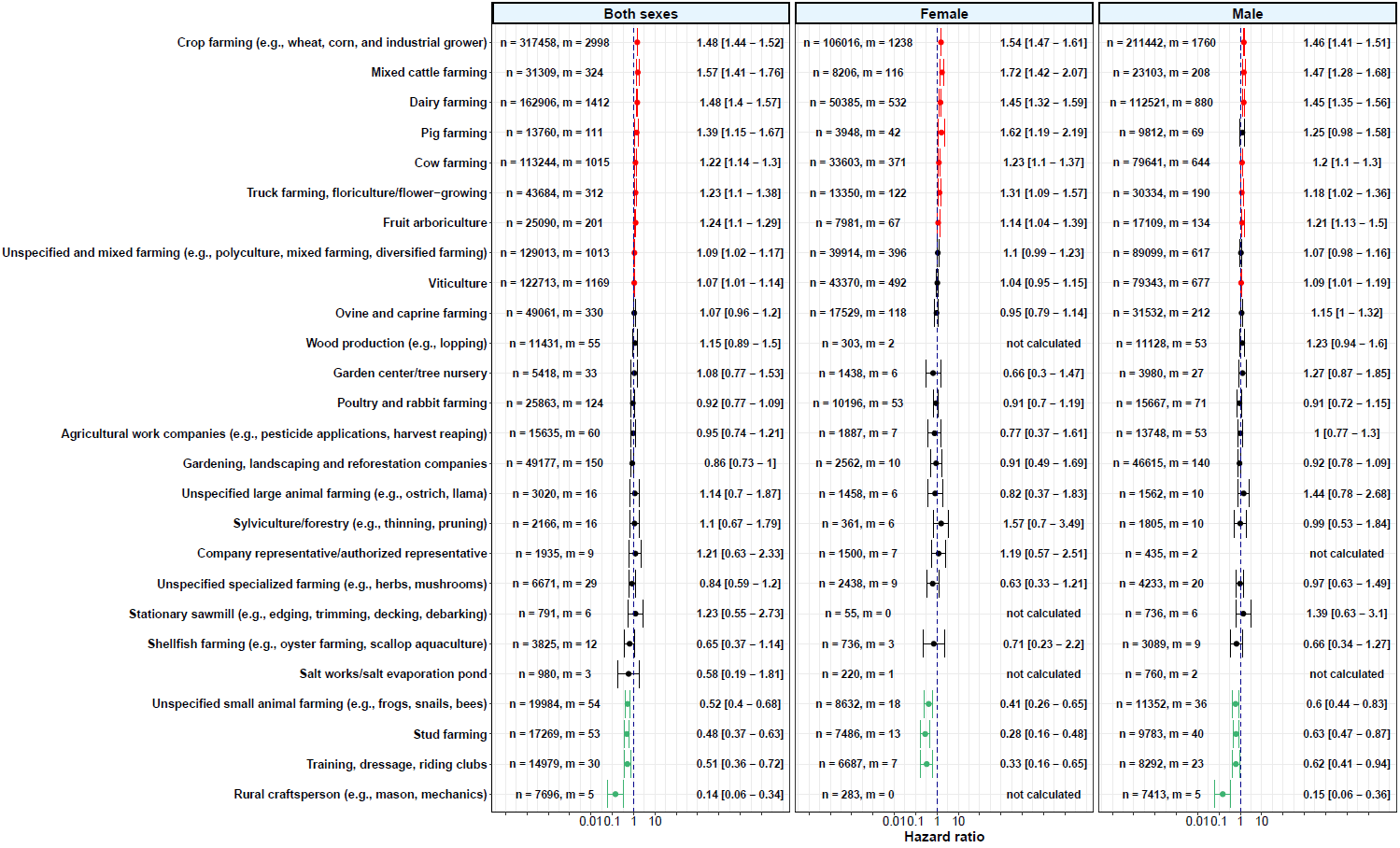


# **Supplementary Figure 16**: Farming activities and risks of Parkinson’s disease, TRACTOR project, 2012-2016 – sensitivity analysis 16

Multivariable Cox regression models for Parkinson’s disease according to each agricultural activity (y-axis) are displayed when the number of exposed cases was sufficient (m ≥ 3). The hazard ratio is represented by a point (x-axis), while error bars represent the 95% confidence interval. The red error bars refer to a higher risk of Parkinson’s disease while the green error bars represent a lower risk of Parkinson’s disease. The black error bars indicate situations where there is no difference in risk of Parkinson’s disease among the farm managers engaged in the considered activity compared to the population of farm managers not performing the considered activity. All analyses were adjusted for sex (for “both sexes” only), age, smoking, first year of the farm’s establishment, median farm surface, number of associates, unemployment status, total number of farms, family status, partner work status, farm location, number of comorbidities, and performing a secondary farming activity. n, number of exposed farm managers; m, number of exposed Parkinson’s disease cases.

## *Sensitivity analysis 17 – control of other diseases not related to farming exposure*

To ensure that any observed effect may be specific to the exposure being studied (i.e., farming-related factors potentially associated with Parkinson’s disease) and not confounded by general health or morbidity, SA17 was restricted to farm managers (non-PD cases) with no known disease or only those with at least one of the following health conditions:

- Metabolic disorders (ICD-10 codes: E70-E88; LTI #17)
- Diabetes mellitus (ICD-10 codes: E08-E13; LTI: #8)
- Overweight, obesity, and other hyperalimentation (ICD-10 codes: E65-E68)
- Schizophrenia (ICD-10 codes: F20-F29)
- Diseases of the eye and adnexa (ICD-10 codes: H00-H59)
- Diseases of the ear and mastoid process (ICD-10 codes: H60-H95)
- Hypertension (ICD-10 code: I10; LTI #12)
- Coronary and ischemic heart diseases (ICD-10 codes: I20-I25; LTI: #3, 5 and 13)
- Cerebrovascular disease (ICD-10 codes: I60-I69; LTI: #1)
- Arthrosclerosis (ICD-10 code: I70)
- Anemia (ICD-10 codes: D63, D64)
- Gout (ICD-10 code: M10)
- Psoriasis (ICD-10 code: L40)
- Hepatitis (ICD-10 codes: B15-B19)
- HIV (LTI #7)
- Hypercholesterolemia (≥ 3 drug reimbursements; ATC codes: C10AA, C10AB, C10B)
- Hemoglobinopathies, chronic constitutional, or severe acquired hemolysis (LTI #10)
- Hemophilias or severe constitutional disorders of hemostasis (LTI #11)
- Cystic fibrosis (LTI #18)
- Severe chronic kidney disease or primary nephrotic syndrome (LTI #19)
- Medullary insufficiency or other chronic cytopenias (LTI #2)
- Progressive idiopathic structural scoliosis (LTI #26)
- Organ transplant consequences (LTI #28)
- Complicated bilharziasis (LTI #4)
- Chronic active liver disease or cirrhosis (LTI #6)
- No known disease

In summary, SA17 aimed to ensure that the farm managers without Parkinson’s disease might exhibit other health conditions that are unrelated to the exposure under investigation. This approach may help clarify whether the exposure has a specific influence on Parkinson’s risk, as opposed to a general health effect that might predispose individuals to various unrelated diseases. Nonetheless, while these unrelated diseases were carefully selected based on expert judgment, their inclusion may have introduced other biases or confounding factors into the model that are challenging to evaluate. The criteria for identifying Parkinson’s disease cases remained the same as in the primary analysis.

Results from SA17 showed that the highest-risk group included FMs engaged in dairy farming, mixed cattle farming, pig farming, crop farming, fruit arboriculture, cow farming, truck farming, unspecified and mixed farming, wood production, and tree nursery (Supplementary Table 4, Supplementary Figure 17). A positive trend was also observed for ovine and caprine farming, viticulture, poultry and rabbit farming, and gardening, landscaping and reforestation companies. By contrast, the lowest-risk group included FMs engaged in small animal farming, training, dressage and riding clubs, stud farming, and rural craftsperson. Tree nursery, stud farming and gardening, landscaping and reforestation companies were the only farming activities exhibiting a sex difference, with male FMs having a higher risk than females.


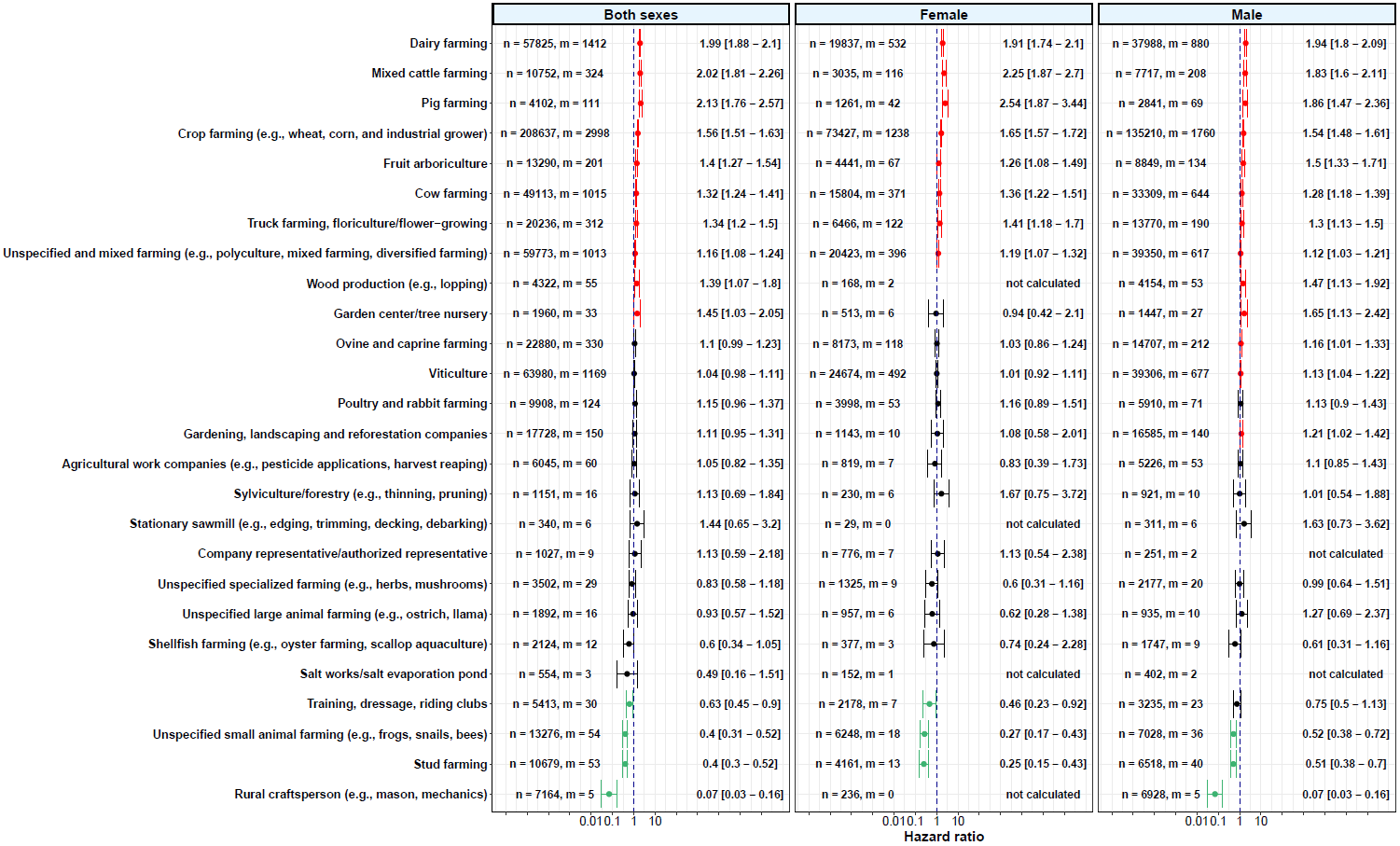


# **Supplementary Figure 17**: Farming activities and risks of Parkinson’s disease, TRACTOR project, 2012-2016 – sensitivity analysis 17

Multivariable Cox regression models for Parkinson’s disease according to each agricultural activity (y-axis) are displayed when the number of exposed cases was sufficient (m ≥ 3). The hazard ratio is represented by a point (x-axis), while error bars represent the 95% confidence interval. The red error bars refer to a higher risk of Parkinson’s disease while the green error bars represent a lower risk of Parkinson’s disease. The black error bars indicate situations where there is no difference in risk of Parkinson’s disease among the farm managers engaged in the considered activity compared to the population of farm managers not performing the considered activity. All analyses were adjusted for sex (for “both sexes” only), age, first year of the farm’s establishment, median farm surface, number of associates, unemployment status, total number of farms, family status, partner work status, farm location, number of comorbidities, and performing a secondary farming activity. n, number of exposed farm managers; m, number of exposed Parkinson’s disease cases.

# **References**

1. Elbaz, A., & Moisan, F. The scientific bases to consider Parkinson’s disease an occupational disease in agriculture professionals exposed to pesticides in France. *J. Epidemiol. Community. Health.* **70** (4), 319-321 (2016).
2. Aloizou, A. M. et al. Pesticides, cognitive functions and dementia: A review. *Toxicol. Lett.* **326**, 31-51 (2020).
3. Bjørklund, G., Dadar, M., Chirumbolo, S., & Aaseth, J. The Role of Xenobiotics and Trace Metals in Parkinson’s Disease. *Mol. Neurobiol.* **57** (3), 1405-1417 (2020).
4. Guillien, A. et al. Prevalence and risk factors for COPD in farmers: a cross-sectional controlled study. *Eur. Respir. J*. **47** (1), 95-103 (2016).
